# *In vitro* models of cancer‐associated fibroblast heterogeneity uncover subtype‐specific effects of CRISPR perturbations

**DOI:** 10.1002/1878-0261.70153

**Published:** 2025-10-27

**Authors:** Elysia Saputra, Shamsudheen Karuthedath Vellarikkal, Lixia Li, Hong Sun, Khoa Nguyen, Amber Montano, Suchitra Natarajan, Federica Piccioni, Alex Michael Tamburino, Xin Yu, Aleksandra Katarzyna Olow

**Affiliations:** ^1^ Department of Data, AI, and Genome Sciences Merck & Co., Inc. Rahway NJ USA; ^2^ Department of Discovery Oncology Merck & Co., Inc. Rahway NJ USA

**Keywords:** CAF heterogeneity, cancer‐associated fibroblast, pancreatic tumor, Perturb‐seq, single‐cell transcriptomics, tumor microenvironment

## Abstract

Cancer‐associated fibroblasts (CAFs) are sought after as potential therapeutic targets due to their pro‐ and antitumorigenic functions, which are attributed to specializations in CAF subtypes. A precise targeting of specific subtypes would be required to design therapies that effectively modulate CAF phenotypes, necessitating translatable model systems to support target discovery efforts. However, not only is our knowledge of CAF heterogeneity in solid tumors lacking, particularly in pancreatic tumors, but the translatability of CAF models has also not been rigorously evaluated. Here, we develop a coculturing model with primary CAFs and immortalized tumor cell lines that can reliably represent CAF phenotypes observed in tumors, with correlations to immuno‐resistant and immunomodulatory phenotypes. Using single‐cell transcriptomics, we characterize the CAF subtype heterogeneity in the *in vitro* CAF cell lines isolated from pancreatic cancer patients and investigate the impact of perturbing potential stromal genes on different CAF subtypes. We also infer the continuum of state changes underlying the interconvertibility of CAF subtypes. Finally, we use immortalized CAF cell lines to perform single‐cell CRISPR perturbations of stromal targets, revealing the subtype‐specific effects of perturbations and the impact of model‐type selection on the translatability of insights.

## Graphical abstract

Development of therapies targeting cancer‐associated fibroblasts (CAFs) necessitates preclinical model systems that faithfully represent CAF–tumor biology. We established an *in vitro* coculture system of patient‐derived pancreatic CAFs and tumor cell lines and demonstrated its recapitulation of primary CAF–tumor biology with single‐cell transcriptomics and phenotypic validation. With Perturb‐seq, we showed that the model enabled studies on subtype‐specific effects of genetic perturbations.

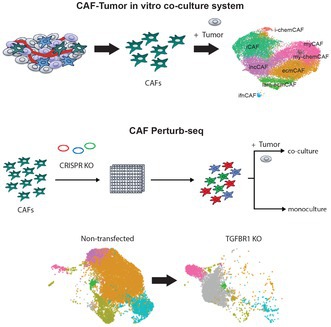

**Abbreviations**
CAF: cancer‐associated fibroblast
DEG: differentially expressed genes
ECM: extracellular matrix
ecmCAF: ECM‐like CAF
FACS: fluorescence‐activated cell sorting
FAP: fibroblast activation protein‐α
FPKM: Fragments Per Kilobase of transcript per Million mapped reads
hTERT: human telomerase reverse transcriptase
iCAF: inflammatory CAF
i‐chemCAF: inflammatory chemokine‐expressing CAF
IFN: interferon
ifnCAF: interferon signaling CAF
IL: interleukin
inCAF: CAF expressing components of innate immune system
KO: knockout
lam‐ecmCAF: laminin‐rich ECM‐like CAF
lncCAF, lnc‐ecmCAF: subtypes of long noncoding RNA‐rich ECM‐like CAF
MST: minimum spanning tree
myCAF: myofibroblastic CAF
my‐chemCAF: myofibroblastic chemokine‐expressing CAF
my‐ecmCAF: myofibroblastic ECM‐like CAF
NP: nonperturbed
NT: nontransfected
PDAC: pancreatic ductal adenocarcinoma
prolifCAF: proliferative CAF
scRNA‐seq: single‐cell RNA sequencing
TME: tumor microenvironment
UMAP: Uniform Manifold Approximation and Projection

## 1 Introduction

The influence of the tumor microenvironment (TME) on cancer progression and therapeutic response is well recognized. Nonmutant cells in the TME, including stromal cells, modulate tumor biology through a plethora of signaling pathways and cross talks with the immune system. Much interest has been given particularly to cancer‐associated fibroblasts (CAFs), a subtype of stromal cells, as potential therapeutic targets due to their vast abundance and highly heterogeneous functions [1]. CAFs are known to regulate extracellular matrix (ECM) remodeling, angiogenesis, growth factor production, inflammatory response, and recruitment of immune cells [1, 2]. In some malignancies such as pancreatic ductal adenocarcinoma (PDAC), a leading cause of cancer‐related mortalities [3], the stroma compartment can constitute the bulk of the tumor [4]. Importantly, CAFs have also been found to correlate with treatment outcome [5] and confer resistance to immunotherapy [6].

CAF targeting has become an attractive strategy for cancer control, giving rise to numerous clinical trial efforts that aimed to reduce CAF activation, modulate CAF activity, or revert CAFs to normal fibroblasts [1]. However, clinical modulations of CAF phenotypes remain challenging due to their biological complexity. Preclinical findings have shown that improper targeting of CAFs may not inhibit tumor growth because different CAF properties may confer pro‐ or antitumorigenic effects [1]. Additionally, CAF biology can vary across tumor lineages. Overall, targeting the stroma is more analogous to targeting immune diseases than tumor‐intrinsic targets, necessitating a deep understanding of CAF phenotypes.

The heterogeneous functions of CAFs arise from specializations in CAF subtypes [1]. In human PDAC and breast cancer, two major CAF subtypes have been characterized: the ‘myofibroblastic’ CAFs (myCAFs) that displayed high levels of αSMA and the ‘inflammatory’ CAFs (iCAFs) that expressed high levels of inflammatory markers [7, 8]. Tumor‐proximal myCAFs regulate ECM contractility and can have tumor‐limiting or tumor‐promoting effects, while tumor‐distal iCAFs secrete immunomodulatory factors. These phenotypes have been hypothesized to be interconvertible [9]. Single‐cell technology has enabled investigations of CAF heterogeneity at a finer scale, revealing more extensive specializations of subtypes. Two recent studies used single‐cell RNA sequencing (scRNA‐seq) and highly multiplexed imaging or flow cytometry on primary breast cancer CAFs to annotate multiple new subtypes, including vascular CAFs, interferon (IFN)‐response CAFs, tumor‐like CAFs, dividing CAFs, reticular‐like CAFs, and CAFs with characteristics of TGF*β* signaling, interleukin (IL) signaling, and the ECM [10, 11]. Some of these subtypes were specializations of the myCAF and iCAF phenotypes, with different associations with immunosuppressive, immuno‐protective, and immuno‐resistant characteristics. For example, the abundance of ECM‐like myCAFs and TGF*β* myCAFs were correlated with immuno‐suppression, whereas myCAFs with wound‐healing characteristics were correlated with immuno‐protection and elevated T‐lymphocyte infiltration [11]. These findings underscore the importance of understanding CAF heterogeneity to target CAFs with high precision.

In this regard, high‐resolution, single‐cell perturbation technologies like Perturb‐seq [12] would be appropriate for identifying targets in highly heterogeneous systems such as the stroma. To facilitate such efforts, it is essential to have an effective and translatable stroma model that can be utilized with longevity. To model CAF–tumor cross talks *in vitro*, CAF–tumor coculture cell line models have been developed [13], but they have not been comprehensively characterized for translational representation. The subtype diversity detected in patient‐derived CAF cell lines could vary across samples due to biological or technical factors, confounding the reliability of scientific findings from these models. Additionally, while immortalization could prolong the lifespan of cell line models, the phenotypic impact of immortalization on CAF cell line properties is unknown.

Here, we used single‐cell technologies to characterize CAF heterogeneity in *in vitro* CAF models and uncover subtype‐specific effects of target knockouts. Using CAFs isolated from four pancreatic tumors, we developed activated CAF cell line models either by coculturing the CAFs with the PDAC cell line BxPC3 or treatment with TGF*β*1, a well‐established CAF‐activating pathway [14]. We then used scRNA‐seq to characterize the phenotypic diversity of the cell lines, their preservation in immortalized models, plasticity trajectories, and recapitulation of immuno‐resistant phenotypes. Our findings highlighted that CAF–tumor coculturing was an effective strategy for engendering the subtype diversity detected in the clinic, even from CAF samples that lacked certain phenotypes at baseline, and revealed the feasibility of preserving this heterogeneity with immortalization. With functional characterization, we validated that the CAF–tumor coculture models demonstrated relevance in promoting tumor growth, enhancing cytokine and ECM production, and increasing contractility. Finally, we used immortalized CAF cell lines to perform Perturb‐seq to investigate the mechanistic changes and subtype‐specific effects of perturbing novel stromal targets, demonstrating the utility of single‐cell perturbation technologies for designing potential CAF modulation strategies.

## 2 Materials and methods

### 2.1 CAF isolation from human tumor tissue

Primary pancreatic tumors from four human donors were purchased from MT Group (detailed information provided in Table S1). All samples were collected under written informed consent according to the standards of the Declaration of Helsinki. We precoated the 10‐cm tissue culture dish with EmbryoMax® 0.1% gelatin solution and incubated it for 30 min at 37 °C. The solution was then replaced with 12 mL FGM‐2 BulletKit media. Tumor samples were fragmentized and digested at 37 °C for 30 min with rotation in 10 mL RPMI 10% FBS with collagenase and DNAsel. The tumor digest was then filtered with a 70 μm filter. The filter was washed with fresh RPMI to add to the collection tube. Repeating this process until tumor fragments disappeared, the collected sample was spun at 300 g for 5 min. After removing the supernatant, the cell pellets were resuspended in 1 mL FGM‐2 BulletKit media, added to the 10 cm dish, and incubated at 37 °C for 48 h. Media containing dead cells and debris were discarded, and the remaining cells were washed twice with 10 mL PBS. With daily replenishment of 12 mL fresh media, colonies of fibroblasts would appear after 7–10 days. After the colonies reached 70% confluency, the culture was expanded in T175 flasks, and the presence of fibroblasts was confirmed by flow cytometry to detect expression of fibroblast activation protein‐α (FAP).

### 2.2 Cell line authentication

The BxPC3 (RRID: CVCL_0186) cell line was obtained from the Merck Cell Bank. BxPC3 authentication was performed by Analytical Biological Services, Inc. in the past three years. To test for *Mycoplasma* contamination, a Polymerase Chain Reaction (PCR) method using primers hybridized with a conserved region in the *Mycoplasma* genome was used. The PCR products were measured with an Agilent 2100 Bioanalyzer and compared with positive and negative controls. The method was used to detect all common strains of *Mycoplasma*, with a minimum detectable concentration of 10 CFU·mL^−1^. All experiments were conducted with mycoplasma‐free cells. Cell species PCR was performed using a PCR method using primers hybridized with 4 species‐specific loci present in human, mouse, rat, and Chinese hamster genomes, in a multiplexed reaction with all primers. PCR products were measured using an Agilent 2100 Bioanalyzer, and evaluated with positive control samples containing DNA of the 4 species as well as negative samples.

### 2.3 *In vitro* CAF activation, immortalization, and sequencing

Parental CAFs (‘donor‐1’, ‘donor‐2’, ‘donor‐3’, ‘donor‐4’) isolated from human tumors were treated with 10 ng·mL^−1^ TGF*β*1 to create TGF*β*1‐treated CAFs (‘TGF*β*1‐donor‐1’ to ‘TGF*β*1‐donor‐4’), and a 1:1 ratio of BxPC3 and CAFs was used to create BxPC3‐CAF cocultures (‘BxPC3‐donor‐1’ to ‘BxPC3‐donor‐4’). The treated cells were cultured in 6‐well collagen‐coated plates (Greiner, Cat # 655956, rat tail collagen type I) for 48 h. The cells were then harvested with 0.05% Accutase (trypsin). For BxPC3‐CAF cocultures, fluorescence‐activated cell sorting (FACS) was used to sort live cells and identify *EPCAM*‐negative cells. To create human telomerase reverse transcriptase (hTERT)‐immortalized BxPC3‐CAF cocultures, we first immortalized the parental CAFs using hTERT cell immortalization (‘hTERT‐donor‐1’ to ‘hTERT‐donor‐4’) and then cocultured the resulting CAFs with BxPC3 with a 1:1 ratio ((‘hTERT‐BxPC3‐donor‐1’ to ‘hTERT‐BxPC3‐donor‐4’)).

### 2.4 scRNA‐seq experiment

Droplet‐based single‐cell partitioning and scRNA‐seq libraries were generated using the 10X Chromium single‐Cell 3′ reagent v3.1 and 5′ reagent v2 kit per the manufacturer's protocol. Briefly, a single‐cell suspension at around 1000 cells·μL^−1^ was mixed with 10× RT reaction master mix and loaded together with gel beads and partitioning oil into a 3′ or 5′Chip. The chip was then loaded onto a Chromium controller for single‐cell GEM generation and barcoding. RNA transcripts were captured by poly(dT) primers and barcoded with cell barcodes and unique molecular identifiers. After emulsion disruption, cDNA molecules were amplified, followed by fragmentation, adapter, and sample indices addition. The final libraries were quantified with Qubit assay, examined by Agilent TapeStation, and sequenced by Illumina NovaSeq 6000.

### 2.5 Perturb‐seq experiment

The sgRNAs used in this work were synthesized by the Functional Genomics Consortium and amplified and inserted into an All‐in‐One vector, pRDA_208 vector, obtained from the Genetic Perturbation Platform at the Broad Institute (vector construct in Fig. S1). The guide sequences are shown in Table S2. Lentiviral infection was used to deliver the guide RNAs into hTERT‐donor‐1 cells. After puro selection and expansion, CAFs were set up either as monocultures or cocultures with BxPC3. For the monocultures, 2% FBS RPMI was used as media. For the BxPC3 cocultures, a 1:1 ratio of BxPC3 to hTERT‐donor‐1 was used. After 2‐day incubation, cells were collected and loaded to the 10× Genomics 5′ High Throughput chip, followed by 10× 5′ CRISPR protocol according to the manufacturer‐supplied protocol [12].

### 2.6 Genomics data preprocessing

10× Genomics Cell Ranger v7.0.1 (RRID: SCR_017344) [15] was used to preprocess both the scRNA‐seq and Perturb‐seq data. First, the default ‘mkfastq’ function was used to demultiplex sequencing reads. Then, the ‘count’ function was used to generate gene expression matrices against the *hg38* genome (annotation GRCh38 v3.0.0). The ‘—include‐introns true’ flag was used to include intron reads.

### 2.7 scRNA‐seq analysis

ScRNA‐seq analysis was performed using *Seurat* 4.1.1 (RRID: SCR_016341) [16] in R. We first performed QC for the 12 nonimmortalized CAF libraries (retaining cells with a number of features between 200 and 9000, and <5% mitochondrial genes). We then used the sctransform method [17] to normalize gene expression, integrated the libraries using Harmony [18], and performed Louvain community detection. Potential tumor cell contaminants (clusters expressing epithelial markers) were removed (Fig. S2). The QC for hTERT‐immortalized versus parental tumor‐cocultured CAF libraries was performed with the same procedure (Fig. S3).

To identify subtypes, the resolution for Louvain community detection was optimized in conjunction with cluster identification. For each resolution, we evaluated whether strongly ‘identifiable’ clusters were produced based on the expression of known CAF subtype markers (Fig. S4) and functional enrichments across different clusters. To compute functional enrichments of every cluster, we computed the significantly upregulated genes (Benjamini–Hochberg adjusted *P*‐value ≤0.01, average log_2_ fold change ≥0.5) [19] and identified significantly enriched Reactome (RRID: SCR_003485) pathways [20] using Fisher's exact test (Benjamini–Hochberg adjusted *P*‐value ≤0.01).

Differentially expressed genes from treatment were identified using the ‘MAST’ method (Benjamini–Hochberg adjusted *P*‐value ≤0.01, absolute average log_2_ fold change ≥1). Reactome pathway enrichments were computed using Fisher's exact test (Benjamini–Hochberg adjusted *P*‐value ≤0.01).

Trajectory analysis was performed using the *TSCAN* package [21] in R. We first computed the centroid coordinates of each cluster and calculated the minimum spanning tree (MST) across the centroids. Given the MST, we obtained the ‘temporal’ ordering of the single cells by projecting each cell to the nearest edge and then determined the pseudotime by computing the distance of the projected coordinate from the ‘root’ node. Finally, DEGs that were significantly correlated with each trajectory were identified (FDR ≤0.001, absolute log fold change ≥0.01).

### 2.8 Perturb‐seq analysis

Cells containing single guides detected by the Cell Ranger CRISPR workflow were used for downstream analysis. The postprocessing of single‐cell transcriptomic count matrices was performed using *Seurat*. We filtered out low‐quality cells, cells with mitochondrial fraction ≥10%, cells transfected by multiple types of gRNA, and cells expressing epithelial markers. The counts were subsequently normalized using sctransform, and batch effects across pools were removed using Harmony (Figs S5, S6).

To identify cells that escaped perturbation, we used the mixscape method [22] in *Seurat*. First, we computed the local perturbation signatures of each transfected cell, taking its 20 nearest ‘nontransfected’ (NT) neighbors as reference. Mixscape then used these perturbation signatures to distinguish successfully perturbed cells (‘KO’) from ‘nonperturbed’ (‘NP’) cells that escaped perturbation using a Gaussian mixture model. The NP cells were removed from downstream analysis (Figs S7, S8).

To cross‐reference the subtypes in the Perturb‐seq data with the original annotations from the scRNA‐seq data, we integrated the two datasets using *Seurat*, setting the scRNA‐seq data as ‘reference’ and the Perturb‐seq data as ‘query’. Single‐cell anchors between the two datasets were identified with reciprocal PCA projection and then used as a parameter to transfer subtype information from reference cells to query cells.

Subtype identification for the Perturb‐seq data was performed using similar optimization metrics as the scRNA‐seq analysis. The selection of optimal clustering parameters was also informed by the purity of each cluster for transferred labels from the scRNA‐seq reference (Fig. S9D).

Compositional differential abundance across subtypes from target perturbation was inferred using scCODA [23]. For each target, the transfection status was used as the binary covariate that affected compositions. We performed a bootstrapped analysis assigning a different reference subtype in each run, as recommended by the developers of scCODA. If no credible effects were detected up to a 0.2 false discovery rate, then there was no significant compositional change.

### 2.9 ELISA

BxPC3 cell lines only (3000 cells·well^−1^), CAF cell lines only (donor‐1 or donor‐5, 3000 cells·well^−1^), and BxPC3 + CAF cocultures (3000 + 3000 cells·well^−1^) were cultured in 100 μL of media per well (DMEM (Gibco 11–965‐092) containing 10% FBS + 1% sodium pyruvate + 1% HEPES + 1% NEAA + 1% Pen/Strep). Then, 60 μL of supernatant was collected after 24 h and 72 h and tested for IL6 and IL11, respectively, by ELISA as per kit manufacturer's instructions (IL‐6 R&D systems cat# D6050/Q‐465859, IL‐11 R&D systems cat# D1100).

### 2.10 Measurements of BxPC3 growth in different treatment conditions

A total of 1500 fluorescence‐labeled BxPC3 cells were seeded in a 96‐well plate by itself or together with different CAF donors at a 1:1 CAF–tumor cell ratio. The coculture and monoculture systems were allowed to grow for 72 h either in the presence or absence of 10 ng·mL^−1^ of TGF‐beta at 37 °C in a Sartorius SX5 IncuCyte. The growth of BxPC3 cells was monitored by capturing their fluorescence signal every 2 h with the IncuCyte system. Image processing and analysis were done by Sartorius IncuCyte software (version 2022B Rev2), and data were graphed with GraphPad Prism 9.

### 2.11 Contractility

CAF cell lines were labeled with CellTrace Yellow (Invitrogen cat# C34567), and BxPC3 cells were labeled with CellTrace Far Red (Invitrogen cat# C34564). A working solution of collagen from a CytoSelect Cell Contraction Kit was prepared according to the manufacturer's instructions (Cell Biolabs cat# CBA‐5021). Following preparation of BxPC3 (125 000 cells·well^−1^), CAF lines (62 500 cells·well^−1^), or BxPC3 + CAF coculture (125 000 + 62 500 cells·well^−1^) in 50 μL of fibroblast media (Lonza cat# CC‐3131, CC‐4126), 200 μL·well^−1^ of the collagen working solution was added to cells. Each well of a contractility plate was filled with 250 μL of the collagen + cell solution. The plate was incubated at 37 °C for 2 h to allow the collagen matrix to solidify. Then, 250 μL of fibroblast media was slowly added to wells so that the collagen matrix would float. The plate was incubated at 37 °C for 48 h and then imaged via Opera Phenix (5× objective, Alexa 568, Alexa 647). Disc diameter measurements were taken at 3–5 measurements per disc. Contractility was analyzed by the average of disc diameter measurements per cell group/condition.

## 3 Results

### 3.1 *In vitro* tumor‐CAF coculture models recapitulate CAF activation and heterogeneity in primary tumors

**Fig. 1:**
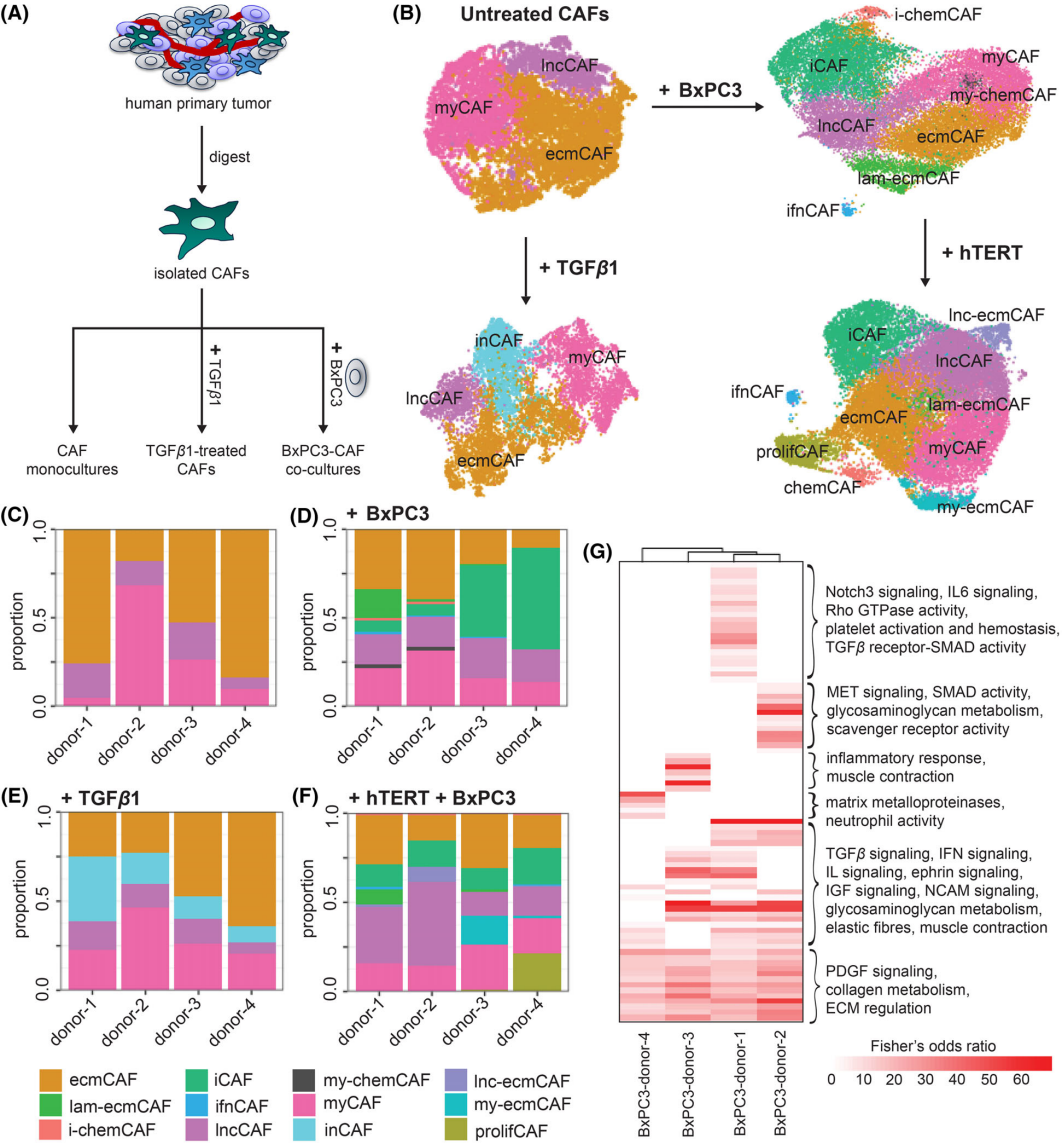
Characterization of *in vitro* cancer‐associated fibroblast (CAF) activation models with single‐cell RNA sequencing. (A) Diagram showing the experimental protocols for establishing *in vitro* CAF models. CAFs were isolated from four primary pancreatic tumors (donor‐1, donor‐2, donor‐3, donor‐4). (B) UMAP visualizations of untreated, TGF*β*1‐CAFs, BxPC3‐CAFs, and hTERT‐immortalized BxPC3‐CAFs. Each UMAP was produced from n = 4 donors. (C) Subtype proportions across untreated CAFs, (D) BxPC3‐CAFs, (E) TGF*β*1‐CAFs, and (F) hTERT‐BxPC3‐CAFs. (G) Heatmap showing the significantly upregulated Reactome pathways from BxPC3 coculturing (Benjamini–Hochberg adjusted *P*‐value ≤0.01). For panels C–G, single‐cell RNA sequencing of each cell line was done with *n* = 1 technical replicate. ecmCAF, ECM‐like CAF; iCAF, inflammatory CAF; i‐chemCAF, inflammatory chemokine‐expressing CAF; ifnCAF, interferon signaling CAF; inCAF, CAF expressing components of the innate immune system; lam‐ecmCAF, laminin‐rich ECM‐like CAF; lncCAF and lnc‐ecmCAF, subtypes of long noncoding RNA‐rich ECM‐like CAF; myCAF, myofibroblastic CAF; my‐chemCAF, myofibroblastic chemokine‐expressing CAF; my‐ecmCAF, myofibroblastic ECM‐like CAF; prolifCAF, proliferative CAF; UMAP, Uniform Manifold Approximation and Projection.

To generate CAF cell lines, we first digested four patient‐derived pancreatic tumor samples (comprising one primary neuroendocrine, one primary adenocarcinoma, and two PDAC tumors) and isolated the fibroblasts, confirmed by the expression of FAP. The four CAF cell lines (‘donor‐1’, ‘donor‐2’, ‘donor‐3’, and ‘donor‐4’) were either kept as monocultures or activated by two methods: treating the CAFs with 10 ng·mL^−1^ of TGF*β*1 or coculturing them with the BxPC3 PDAC cell line with a 1:1 tumor‐to‐CAF ratio (Fig. 1A). We validated that coculturing primary CAF cell lines with BxPC3 significantly increased the levels of cytokines IL6 after 24 h and IL11 after 72 h, which were indicators of CAF activation, compared to the CAFs or BxPC3 alone (Fig. S10).

We then asked whether *in vitro* activation could produce CAF subtypes observed in primary tumors. We performed scRNA‐seq and graph‐based clustering to identify the subtypes (Fig. S4) in untreated CAFs, TGF*β*1‐treated CAFs, and BxPC3‐CAF cocultures (parental and hTERT‐immortalized) (Fig. 1B). In untreated CAFs, we identified three subtypes: myCAFs, ‘ecmCAFs’ (ECM‐like CAFs), and ‘lncCAFs’ (ecmCAFs expressing long noncoding RNAs). Despite using FAP, an iCAF marker, to confirm the presence of CAFs after isolation from primary tumors, we did not find any iCAFs prior to activation, corroborated by the fact that no clusters expressed high levels of iCAF signatures from primary human and mouse PDAC [24] (Fig. S4A). We also validated that the myCAF cluster expressed the highest levels of primary myCAF signatures (Fig. S4B) and pan‐fibroblast TGFβ response (Pan‐F‐TBRS) signatures (Fig. S4C).

The ecmCAF cluster expressed low levels of αSMA and high levels of ECM markers. The presence of CAFs with strong ECM characteristics has been previously identified in human breast cancers [10, 11]. Our clustering parameters could also segment ecmCAFs based on expression markers of different ECM components (Fig. S11), including the interstitial matrix (‘ecmCAF‐im’, expressing collagens I, III, and V), the basal membrane (‘ecmCAF‐bm’, expressing laminins and collagen IV), and metalloproteinase‐expressing ECM (‘ecmCAF‐mp’). Meanwhile, the top‐ranking markers of the lncCAF cluster were long‐noncoding RNAs (lncRNAs) *MALAT1*, *NEAT1*, *MEG3*, and *CARMN*, which have been characterized in CAF‐released exosomes [25, 26, 27] and regulate CAF–tumor communication and cancer progression [28]. The baseline heterogeneities of the untreated CAF samples were variable (Fig. 1C).

Upon coculturing, new subtypes emerged (Fig. 1B,D). Specifically, BxPC3‐CAF coculturing produced iCAFs, chemokine‐expressing myofibroblastic or inflammatory CAFs (‘my‐chemCAFs’, ‘i‐chemCAFs’), and a small population of CAFs with high IFN activity (‘ifnCAFs’) (Fig. 1B, Fig. S4). The iCAF cluster strongly expressed cytokines (*IL6*, *IL11*, *IL6ST*, *IL7R*), chemokines (*CCL2*, *CXCL1*, *CXCL2*, *CXCL8*, etc.), and proinflammatory molecules (*SOD2*). The chemCAF populations were marked by high expression of chemokines *CCL11* and *CCL2*. The ifnCAF cluster exhibited high expression of the IFNα/β and IFNγ CAF signatures previously annotated in breast cancers [11]. These observations highlighted that coculturing patient‐derived CAFs with a cancer cell line could serve as an effective strategy to give rise to classical CAF phenotypes, even from CAF samples that lacked certain phenotypes at baseline.

While all subtypes were observed in all cell lines, the subtype proportions varied across samples (Fig. 1C–E). The iCAF subtype constituted a large proportion of BxPC3‐donor‐3 and BxPC3‐donor‐4 but was lowly represented in BxPC3‐donor‐1 and BxPC3‐donor‐2. Notably, both donor‐3 and donor‐4 CAFs were collected from PDAC samples, while donor‐1 and donor‐2 CAFs were collected from primary neuroendocrine and primary adenocarcinoma samples, respectively (Table S1). Interestingly, we observed a distinction in the protumorigenic effects of iCAF‐rich versus iCAF‐deficient CAFs. Specifically, coculturing BxPC3 with iCAF‐deficient donor‐2 CAFs resulted in an increase in BxPC3 population, while no increase was observed from coculturing with iCAF‐rich donor‐3 CAFs (Fig. S12). When the BxPC3‐CAF cocultures were further treated with TGF*β*, we observed an increase in BxPC3 population, but the growth was markedly stronger for the iCAF‐rich coculture. Notably, no BxPC3 growth was observed from treating BxPC3 with TGF*β* alone. We also validated that coculturing BxPC3 with the CAF cell lines resulted in contraction of the cells, but the iCAF‐deficient lines noticeably caused a stronger contraction than the iCAF‐rich line (Fig. S13). Meanwhile, a laminin‐expressing ecmCAF subtype (‘lam‐ecmCAF’) was mainly found in BxPC3‐donor‐1. In contrast, TGF*β*1 treatment did not significantly change subtype heterogeneity. However, we identified a new subtype expressing components of the innate immune system (‘inCAF’ (Fig. 1B)), which did not express high levels of iCAFs, myCAFs, and Pan‐F‐TBRS signatures (Fig. S4D–F).

We next evaluated whether the heterogeneity of BxPC3‐CAF cocultures could be preserved through hTERT‐immortalization. We found that all the subtypes identified in the parental lines were also present in the immortalized lines (Fig. 1B,F; Fig. S14B,C), although their compositions were different (Fig. 1D,F). We also highlighted some hTERT‐induced changes in ecmCAFs. Specifically, a population with strong myCAF properties that also expressed collagen V (‘my‐ecmCAF’) appeared in hTERT‐BxPC3‐donor‐3 and hTERT‐BxPC3‐donor‐4; a population with strong proliferative characteristics (‘prolifCAF’) appeared in hTERT‐BxPC3‐donor‐4; and a population expressing lncRNAs, laminins, and collagens (‘lnc‐ecmCAF’) appeared across all lines but mostly in hTERT‐BxPC3‐donor‐2 (Figs. S14B,C). Overall, hTERT‐BxPC3‐donor‐1 was the least affected by hTERT‐induced changes.

Finally, we investigated the underlying mechanisms that drove CAF activation in these *in vitro* models. We identified differentially overexpressed genes from CAF activation in a sample‐specific manner (Figs S15, S16) and computed the enrichment of Reactome pathways in these genes (Fig. 1G, Fig. S15E,F). We found a strong consistency in pathways upregulated from both *in vitro* CAF activation strategies. Notably, the upregulated pathways comprised a diverse collection of pathways known to drive CAF activation, including PDGF signaling, IL signaling, TGF*β* signaling, SMAD, and ECM regulation and organization [1]. Some pathways were only upregulated from BxPC3‐CAF coculturing, such as Notch3 signaling and IFN signaling. Notch3 signaling indeed plays a role in CAF activation [29], and IFN signaling likely drove the appearance of ifnCAFs. It is thus likely that BxPC3‐CAF coculturing could capture the reciprocal signaling cross talks between tumor cells and CAFs comprehensively. We also note some sample specificity in these pathway‐level signals, which likely contributed to the variations in heterogeneity profiles of the resulting CAF populations. Overall, BxPC3‐CAF cocultures could effectively emulate transcriptomic signals expected of CAF activation in our *in vitro* model system.

### 3.2 Subtypes in BxPC3‐CAF cocultures correlate with primary CAF phenotypes and immuno‐resistance

**Fig. 2:**
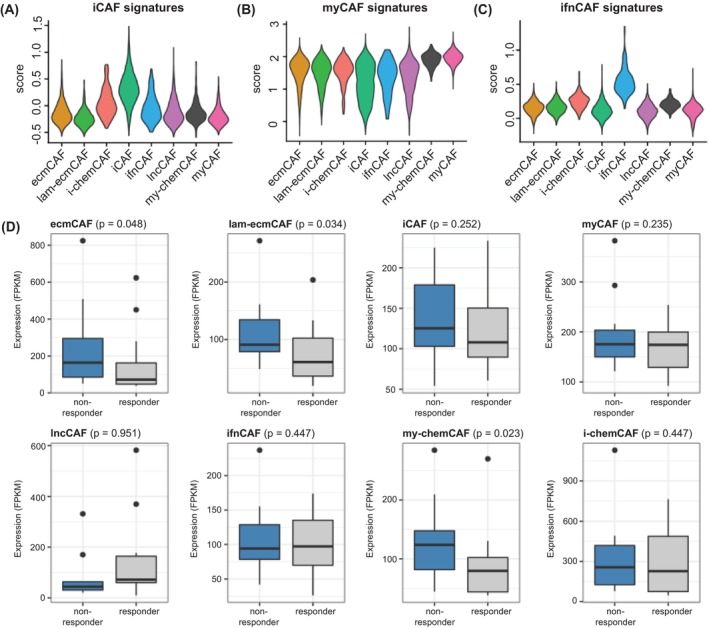
BxPC3‐CAF subtypes correlate with primary CAF subtypes and immuno‐resistant phenotypes. (A) Expression module scores for inflammatory CAF signatures annotated from human and mouse pancreatic ductal carcinoma (PDAC) across BxPC3‐CAF subtypes, and the same plots for (B) myofibroblastic CAF signatures from human and mouse PDACs and (C) interferon CAF signatures from human breast cancer. (D) The expression (in FPKM) of BxPC3‐CAF subtype markers in tumors of 28 melanoma patients who responded (*n* = 14) or did not respond (*n* = 14) to anti‐PD1 treatment. One‐sided Wilcoxon rank‐sum test *P*‐values between the expressions of the markers of each subtype in responders and nonresponders are shown. Boxplot whiskers represent the range of data points excluding outliers, obtained by extending the box by 1.5× the interquartile range. CAF, cancer‐associated fibroblasts; ecmCAF, ECM‐like CAF; FPKM, Fragments Per Kilobase of transcript per Million mapped reads; iCAF, inflammatory CAF; i‐chemCAF, inflammatory chemokine‐expressing CAF; ifnCAF, interferon signaling CAF; lam‐ecmCAF, laminin‐rich ECM‐like CAF; lncCAF, long noncoding RNA‐rich ECM‐like CAF; myCAF, myofibroblastic CAF; my‐chemCAF, myofibroblastic chemokine‐expressing CAF.

Next, we investigated whether the *in vitro* CAF subtypes in BxPC3‐CAF cocultures could reliably represent primary CAF phenotypes in patient studies. We first evaluated the expression of iCAF and myCAF signatures annotated by Elyada et al. [24] from human PDAC and breast primary tumors across our coculture subtypes. We found that the iCAF subtype expressed highly specific, elevated levels of primary iCAF signatures relative to the other subtypes (Fig. 2A). A similar specificity was also observed for the myCAF subtype expressing high levels of primary myCAF signatures (Fig. 2B). Finally, the ifnCAF subtype showed a strong correlation with the ifnCAF subtype signatures defined from human primary breast cancers only [10] (Fig. 2C).

We also asked whether different CAF subtypes were correlated with immunotherapy resistance. As a clinical reference, we used a dataset of metastatic melanoma patients who were treated with anti‐PD‐1 (pembrolizumab) [30]. Among the 28 patients for whom the dataset was available, 14 patients responded partially or completely (‘responders’), while the remaining patients exhibited a progressive disease (‘nonresponders’). Using bulk gene expression measurements in these patients prior to anti‐PD‐1 treatment, we computed the mean expression of signatures of each *in vitro* CAF subtype and computed the association between the mean expression and the nonresponder status of the patients, using a one‐sided Wilcoxon rank‐sum test (Fig. 2D). We found that resistance to anti‐PD‐1 was significantly correlated with high expression of ecmCAF, lam‐ecmCAF, and my‐chemCAF signatures, while no correlation was observed with the remaining subtypes. Thus, the correlation with nonresponse to anti‐PD‐1 and the presence of iCAFs suggested that our BxPC3‐CAF cocultures could potentially model both immune modulatory and immuno‐resistant functions observed in human tumors.

### 3.3 Trajectory analysis elucidates CAF plasticity in BxPC3‐CAF cocultures

**Fig. 3:**
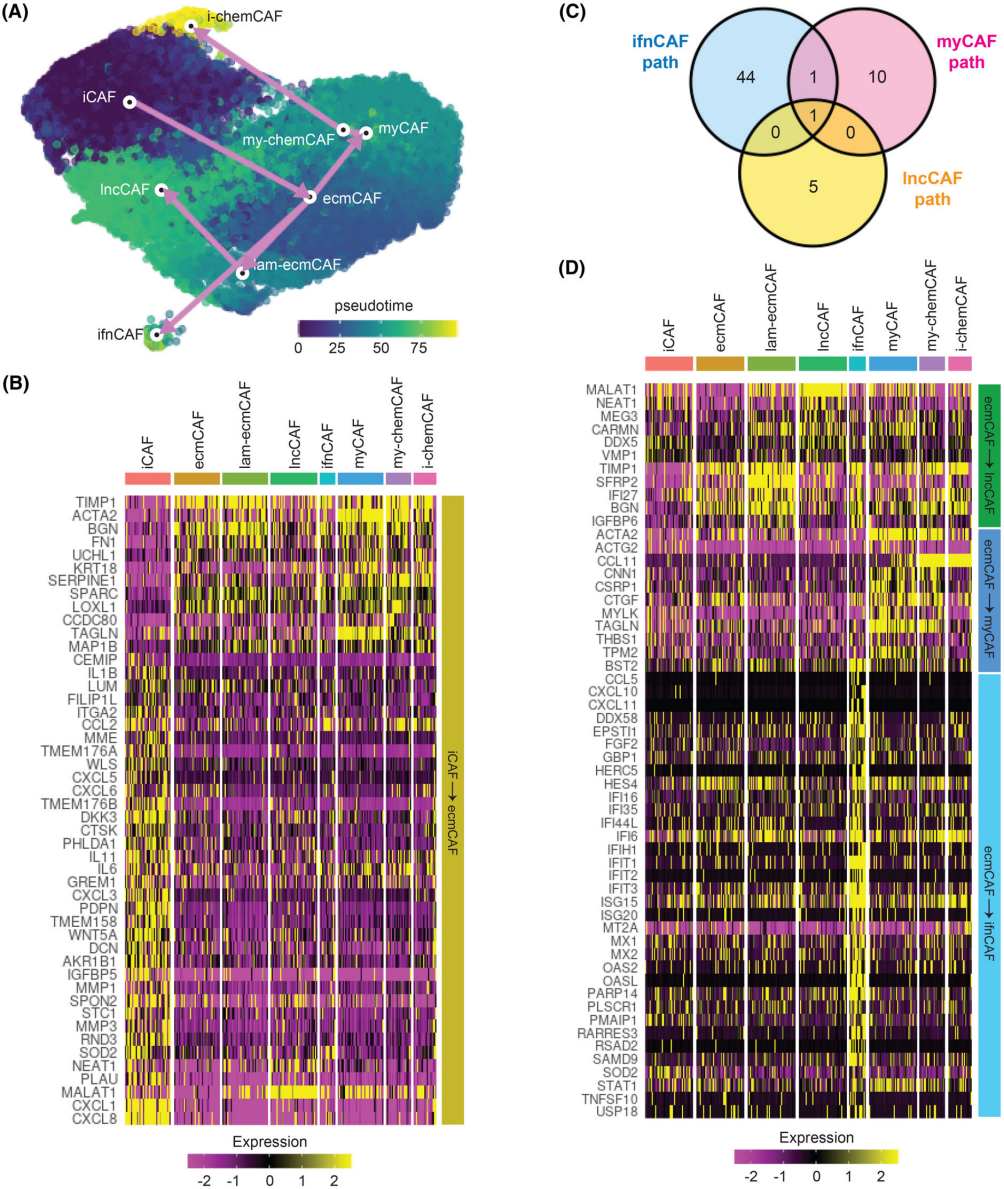
Trajectory analysis reveals plasticity across BxPC3‐CAF subtypes. (A) UMAP visualizations of BxPC3‐CAFs scRNA‐seq data (*n* = 4) annotated with the transitional trajectory and the corresponding pseudotimes. White circles denote the cluster centroids. (B) Heatmap showing the expression of genes significantly associated with the initial transition from iCAFs to ecmCAFs. (C) Venn diagram showing the number of upregulated genes that are significantly associated with the transition from ecmCAFs to ifnCAFs, lncCAFs, and myCAFs (+ chemCAFs). (D) Heatmap showing the expression of genes significantly associated with the transition from ecmCAFs to terminal states. CAF, cancer‐associated fibroblasts; ecmCAF, ECM‐like CAF; iCAF, inflammatory CAF; i‐chemCAF, inflammatory chemokine‐expressing CAF; ifnCAF, interferon signaling CAF; lam‐ecmCAF, laminin‐rich ECM‐like CAF; lncCAF, long noncoding RNA‐rich ECM‐like CAF; myCAF, myofibroblastic CAF; my‐chemCAF, myofibroblastic chemokine‐expressing CAF; UMAP, Uniform Manifold Approximation and Projection.

Previous findings of interconvertibility of CAF subtypes [9, 31] suggest that shifting CAF phenotypes to antitumorigenic subtypes may be a feasible strategy for cancer control. However, the mechanism of this interconvertibility in pancreatic tumors is unknown. To characterize the plasticity among the CAF subtypes, we used the *TSCAN* method [21] to infer the trajectory of subtype transitions and compute the pseudotimes of the BxPC3‐CAF coculture cells. We selected the ‘initial’ state of the CAFs by the expression of ‘universal fibroblast markers’ that were determined from normal and perturbed human tissues, including normal pancreas and PDAC [32]. Based on these markers (Fig. S17), we selected iCAFs as the ‘root’ node for pseudotime computation. This is consistent with the observation in primary breast cancer CAFs in which the iCAF population with detoxification signatures was found to be the transitional state from normal fibroblasts to CAFs [31]. The result shows a trajectory that started with an initial transition from iCAFs to ecmCAFs, followed by a three‐way bifurcation that culminated in lncCAFs, ifnCAFs, and myCAFs, respectively. A small proportion of myCAFs further differentiated into myofibroblastic and inflammatory chemCAFs (Fig. 3A).

We then characterized the mechanisms underlying these trajectories by identifying the differentially expressed genes (DEGs) that were significantly correlated with each trajectory (Benjamini–Hochberg adjusted *P*‐value <0.001). Fig. 3B shows the DEGs that were associated with the iCAF‐to‐ecmCAF transition. We observed an upregulation of myCAF genes that regulated contractility (*ACTA2*, *TAGLN*) and ECM genes that promoted ECM assembly and cell adhesion (*TIMP1*, *BGN*, *FN1*, *SERPINE1*, *LOXL1*, *CCDC80*). In the opposite direction, the downregulated DEGs included a promoter of iCAF formation (*PLAU*), ECM degraders (*MMP1*, *MMP3*, *SOD2*), cytokines (*IL6*, *IL11*, *IL1B*), chemokines (*CXCL8*, *CXCL1*, *CXCL6*, *CXCL5*, *CCL2*), lncRNAs (*MALAT1*, *NEAT1*), and cation channels that regulated antigen presentation (*TMEM176A*, *TMEM176B*). The levels of some DEGs were sustained as the cells transitioned from ecmCAFs to other states (e.g., *CCL2* and myCAF gene levels remained high as ecmCAFs transitioned to myCAFs, my‐ecmCAFs, and i‐chemCAFs), but there were others that reverted as ecmCAFs transitioned to other states (e.g., lncRNAs in the lncCAF path).

From ecmCAFs, the trajectory split into three branches. Most of the upregulated DEGs were privately associated with one path, with *MALAT1* being the only one associated with all paths and *BST2* being associated with the ifnCAF and myCAF paths (Fig. 3C). Meanwhile, a handful of downregulated DEGs were identified for the lncCAF path (*TIMP1*, *SFRP2*, *IFI27*, *BGN*, *IGFBP6*), and none for the ifnCAF and myCAF paths. Fig. 3D shows the expression of the DEGs associated with the three trajectories. Among the DEGs associated with the lncCAF path, we interestingly found that for almost all of them, after the initial changes from the iCAF‐to‐ecmCAF transition, their levels reverted direction as the ecmCAFs transitioned to lncCAFs. Along the myCAF path, we observed the upregulation of myCAF markers, as well as *CCL11* levels that kept increasing as the cells transitioned from myCAFs to my‐chemCAFs and i‐chemCAFs. Finally, along the ifnCAF path, we observed the upregulation of many interferon‐inducible genes (e.g., *IFIT3*, *IFI6*, *OASL*, *ISG15*), chemokines (*CCL5*, *CXCL10*, *CXCL11*), and others.

Overall, our findings suggest that iCAFs as the initial cancerous fibroblast phenotype and ecmCAFs as a ‘reservoir’ of later‐stage phenotypes may serve as plausible anticancer targets. By characterizing the DEGs associated with these subtype transitions, we identified potential modulations that we could test to interconvert CAF subtypes with an anticancer objective.

### 3.4 Perturb‐seq experiments with immortalized CAF cell lines reveal subtype‐specific changes from perturbing stromal targets

**Fig. 4:**
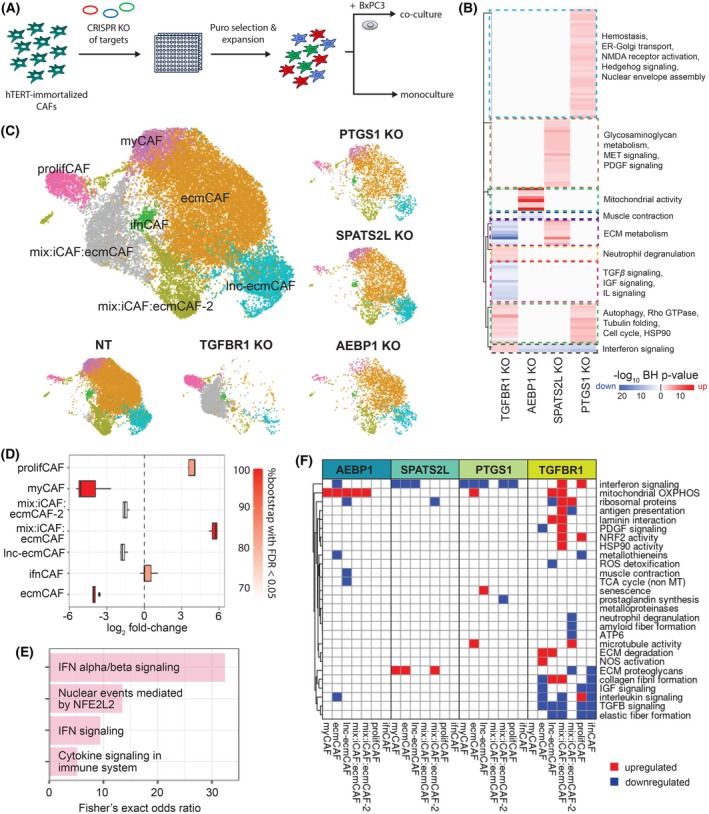
Perturb‐seq experiments of candidate targets with immortalized *in vitro* CAF models. (A) Workflow of Perturb‐seq experiments on hTERT‐BxPC3‐donor‐1 CAFs (*n* = 1 technical replicate). (B) Upregulated and downregulated pathways enriched for differentially expressed genes from perturbation of *TGFBR1*, *AEBP1*, *SPATS2L*, or *PTGS1*. (C) UMAP visualizations of CAF subtypes across treatments. (D) Changes in compositional abundances of CAF subtypes resulting from perturbing *TGFBR1*, computed by bootstrapped experiments with scCODA (false discovery rate ≤ 0.05). Boxplot whiskers represent the range of data points excluding outliers, obtained by extending the box by 1.5 × the interquartile range. (E) Significantly enriched pathways in the mix:iCAF:ecmCAF population (Benjamini–Hochberg adjusted *P*‐value ≤0.01). (F) Pathways that are significantly upregulated or downregulated from perturbing each target (Benjamini–Hochberg adjusted *P*‐value ≤0.01). CAF, cancer‐associated fibroblasts; ecmCAF, ECM‐like CAF; iCAF, inflammatory CAF; ifnCAF, interferon signaling CAF; KO, knockout; lnc‐ecmCAF, long noncoding RNA‐rich ECM‐like CAF; myCAF, myofibroblastic CAF; NT, nontransfected; prolifCAF, proliferative CAF; UMAP, Uniform Manifold Approximation and Projection.

Finally, we used the hTERT‐BxPC3‐donor‐1 coculture and hTERT‐donor‐1 monoculture cell lines to perform single‐cell perturbation experiments with Perturb‐seq [12] on several genetic targets. The targets—*PTGS1*, *SPATS2L*, and *AEBP1*—were selected based on their novelty, upregulation in CAFs, and associations with CAF biology [33, 34], in addition to *TGFBR1* as a positive control‐like gene. The CAFs were either cocultured with BxPC3 at a 1:1 ratio for 48 h or kept as monocultures (Fig. 4A).

Starting with the coculture data, we first used the mixscape method [22] to distinguish between successfully perturbed ‘knockout’ (KO) cells and ‘nonperturbed’ (NP) cells that were successfully transfected but ‘escaped’ perturbation. The KO cells indeed expressed reduced levels of the targets compared to the nontransfected (NT) and NP cells (Fig. S7). After excluding all NP cells, we identified significant DEGs from the perturbations and computed pathway enrichments (Fig. 4B). Interestingly, perturbing *TGFBR1* and *SPATS2L* generally produced opposing effects; perturbing *TGFBR1* upregulated IFN activity and downregulated ECM metabolism, which was the reverse of *SPATS2L*. IFN activity was also downregulated from *AEBP1* and *PTGS1* perturbation. Meanwhile, both *TGFBR1* and *PTGS1* perturbations upregulated pathways related to autophagy, Rho GTPase activity, tubulin folding, cell cycle, and HSP90 activity. There were also pathway signals that were specific to each perturbation. *TGFBR1* perturbation upregulated neutrophil degranulation and downregulated TGF*β* signaling, IGF signaling, and IL signaling; *SPATS2L* perturbation upregulated glycosaminoglycan metabolism, MET signaling, and PDGF signaling; *AEBP1* perturbation downregulated muscle contraction and upregulated mitochondrial activity; and *PTGS1* perturbation upregulated hemostasis, ER‐Golgi transport, NMDA receptor activity, Hedgehog‐off state, and nuclear envelope assembly. Most of the upregulated pathways from *PTGS1* perturbation were driven by *TUBA1A*, *TUBA1B*, and *TUBA1C*, indicating that the biological impact of *PTGS1* perturbation largely involved microtubule regulation.

To evaluate subtype‐specific effects, we first annotated CAF subtypes (Fig. 4C) with high purity of myCAFs, ecmCAFs, ifnCAFs, prolifCAFs, and lnc‐ecmCAFs, while noting the presence of two distinct clusters exhibiting a mix of iCAF and ecmCAF properties. We observed that *TGFBR1* perturbation resulted in the disappearance of ecmCAFs and myCAFs, consistent with *TGFBR1's* role in promoting ECM formation. Interestingly, *TGFBR1* perturbation promoted the expansion of the prolifCAF cluster and the appearance of the mix: iCAF: ecmCAF cluster that was barely present in the NT, *AEBP1*, *PTGS1*, and *SPATS2L* KO groups. We labeled this new cluster as ‘mix:iCAF:ecmCAF’ with reference to subtype labels transferred from the scRNA‐seq data, which assigned ~53% of cells in this cluster as iCAFs and ~35% as ecmCAFs (Fig. S9C). To validate this observation, we performed compositional differential abundance analysis with bootstrapped experiments using the scCODA tool [23] and found that the increase of mix: iCAF: ecmCAFs and the decrease of myCAFs and ecmCAFs were significant in 100% of the bootstrapped runs (Benjamini–Hochberg adjusted *P*‐value ≤0.05) (Fig. 4D).

To characterize the mix:iCAF:ecmCAF population, we identified the DEGs in this cluster and performed pathway enrichment analysis. While the cluster has ECM and inflammatory characteristics, it also showed elevated levels of IFN signaling and *NFE2L2* (NRF2)‐mediated activity (Fig. 4E). As scCODA highlighted no significant change in ifnCAF proportion, the increase in IFN activity from *TGFBR1* perturbation (Fig. 4B) was driven by the appearance of this cluster. The genes involved in the NRF2‐related pathway hits were *NQO1*, *TXNRD1*, *PSMB8*, *PSMB9*, and *G6PD*.

We investigated the transcriptional changes within each subtype induced by perturbing the different targets, by identifying DEGs of the KO versus NT cells. Fig. 4F shows the categories of pathways that were significantly enriched for the perturbation‐induced DEG across different subtypes (detailed list of Reactome pathways in Tables S3–S6). This analysis allowed us to learn the distribution of functional changes that occurred in different subtypes due to the perturbation. While some pathway changes arose due to a specific perturbation, several were observed across perturbations, highlighting the importance of these pathways in undergirding CAF phenotypes. A pervasive change that occurred across different perturbations was the upregulation of mitochondrial genes that were components of oxidative phosphorylation in ecmCAFs with *PTGS1* perturbation, lnc‐ecmCAF and mix:iCAF:ecmCAFs with *TGFBR1* perturbation, and most prominently in myCAFs and multiple subtypes with ecmCAF characteristics with *AEBP1* perturbation. Another pervasive change is the downregulation of interferon signaling across different subtypes due to perturbation of *AEBP1*, *SPATS2L*, and *PTGS1*. An exception to this observation was *TGFBR1* perturbation that upregulated interferon signaling specifically in mix:iCAF:ecmCAFs and prolifCAFs, both of which also showed upregulation of NRF2 activity. A third example is the upregulation of ECM proteoglycans in the myCAF, ecmCAF, and mix:iCAF:ecmCAF‐2 populations due to *SPATS2L* perturbation, which were downregulated by *TGFBR1* perturbation.

Finally, we repeated the Perturb‐seq experiment and analysis on the hTERT‐donor‐1 monoculture. Consistent with the scRNA‐seq findings, we found that the BxPC3‐cocultured CAFs and the CAF monoculture had highly distinct phenotypes before and after perturbation (Figs. S18A,B). The distinction between the two model systems was also apparent in the pathway enrichment signals, noting the higher agreement for *TGFBR1* perturbation (Fig. S18C–F). This observation underscored that the choice of model systems could substantially influence findings in downstream analysis.

## 4 Discussion

In this work, we established an *in vitro* coculture system of patient‐derived CAFs with a cancer cell line that could recapitulate CAF and tumor biology. Using single‐cell technologies, we demonstrated that the model system not only enabled the identification of classical and new pancreatic CAF subtypes, but also provided a tractable system to modulate specific subtypes via Perturb‐seq. While our results were obtained from coculturing CAFs with the BxPC3 cell line, we found that many of the key processes enriched in CAFs post‐tumor coculture were similar across pancreatic cancer cell lines (Fig. S19). BxPC3 was selected instead of other lines because the BxPC3‐CAF coculture resulted in the strongest gene‐level effects, particularly for genes relevant to pan‐disease fibrosis (Fig. S20), which would allow for a robust signal window for downstream studies.

In addition to detecting previously characterized CAF phenotypes in both primary *in vitro* cell cultures and after hTERT‐immortalization, we identified new CAF subtypes that, to our knowledge, had not been previously characterized in pancreatic CAFs. Similar subtypes have been reported in breast cancer [10, 11], including myofibroblasts, inflammatory fibroblasts, and CAFs enriched for ECM‐like characteristics and interferon signaling. However, the expression signatures of these pancreatic subtypes did not correlate with primary breast cancer CAFs (Fig. S21). Indeed, CAFs from different anatomical sources can have context specificity [35, 36, 37] and there is evidence that CAFs mostly originate from local tissue sources [38]. Importantly, while the ability to immortalize CAF–tumor cocultures could prolong the availability of preclinical CAF models with representation of classical CAF subtypes, we note some newly emerging ECM‐like phenotypes that could arise from immortalization in a donor‐specific way. Although the proportions of these emerging phenotypes were minimal in our data, their presence should be noted as they could influence the interpretation of downstream functional experiments.

Despite the weak correlation between breast cancer and pancreatic CAF subtypes, the plasticity trajectories among the subtypes described in this work were analogous to findings from primary breast cancer [31], which outlined that iCAFs with detoxification signatures were the initial transitional state from healthy to malignant fibroblasts, and that this population transitioned to ECM‐myCAFs that eventually branched into myCAFs with different properties. Our predicted trajectory also identified iCAFs as the first diseased state, which then transitioned to ecmCAFs that served as a ‘reservoir’ seeding other subtypes. This observation suggested conserved transitional mechanisms among CAFs in pancreatic and breast cancer. The observation of ecmCAFs as the intermediate state between iCAFs and myCAFs was consistent with the characterization of a ‘quiescent’ state between the two subtypes in PDAC [9]. The identification of genes associated with each state transition could propose potential strategies to modulate CAF subtypes away from the ones associated with immuno‐resistance.

We observed that perturbing *TGFBR1* eradicated ecmCAFs and myCAFs, consistent with the role of TGF*β* signaling in promoting myofibroblast differentiation and ECM dysregulation [39, 40]. However, it also promoted the appearance of a CAF subtype with elevated interferon and NRF2 signaling activities. Mutations of NRF2 in tumor cells that resulted in NRF2 hyperactivity are known to promote oncogenic characteristics [41] and correlate positively with immune evasion [42]. However, the effect of stromal NRF2 on cancer progression is less straightforward. In some mouse models, elevated stromal NRF2 activity could limit the progression of malignant tumors with NRF2 hyperactivity [43] and the microenvironment of NRF2‐deficient mice could promote metastasis [44]. Paradoxically, stromal NRF2 activation by antioxidant antidiabetic tumors has been found to promote metastasis [45]. Future work could elucidate the biology of stromal NRF2 and its effects on cancer progression in different indications.

Mitochondrial oxidative phosphorylation was upregulated across ECM‐like subtypes from *AEBP1*, *PTGS1*, and *TGFBR1* perturbations. Metabolic reprogramming is known as a hallmark of CAFs [46]. Specifically, TGF*β*1 or PDGF activation of CAFs can cause a metabolic shift of CAFs from oxidative phosphorylation to glycolysis, leading to tumor promotion [47]. The increase of mitochondrial oxidative phosphorylation genes in several subtypes suggested that perturbing these targets could reverse the metabolic state of glycolytic CAFs back to oxidative phosphorylation.

We also observed that *SPATS2L* perturbation upregulated pathways related to ECM proteoglycans. The upregulated genes included glycoproteins (*FST*, *CLU*), small leucine‐rich proteoglycans (*DCN*, *LUM*), leucine‐rich repeat protein *PRELP*, and cathepsin K (*CTSK*). In many cancers, tumoral and stromal decorin (*DCN*) has anticancer properties, attributed to its inhibition of cancer‐associated and CAF‐activating pathways including TGF*β*, PDGF, c‐Met, and CTGF signaling [48]. Meanwhile, lumican (*LUM*) overexpression can be anti‐ or protumorigenic in different cancers [49], but it decelerated proliferation in PDAC specifically [50]. This suggests that inhibiting stromal *SPATS2L* can potentially produce antitumor effects by modifying proteoglycan‐ and glycosaminoglycan‐mediated mechanisms of ECM remodeling [49]. Notably, ECM proteoglycans were downregulated in mix:iCAF:ecmCAF‐2 and ifnCAF from *TGFBR1* perturbation, highlighting the necessity for understanding the underlying biology of different CAF phenotypes to design effective stroma‐targeted treatment strategies.

Finally, we acknowledge some caveats of our study. Firstly, we lack information on the subtype diversity of the CAFs originally derived from the donor patients and thus were unable to evaluate whether this diversity was maintained in culture. Secondly, although we used flow cytometry to detect FAP, an inflammatory CAF marker, for confirming the presence of CAFs in the CAFs isolated from tumors, we did not observe a cluster of iCAFs in our scRNA‐seq data at baseline. This observation could have arisen from the original CAF phenotypes, repeated passaging, the type of collagen‐coated plates used for culture, or other factors. Nonetheless, our work illustrated that coculturing CAFs with a cancer cell line could be an effective strategy for inducing CAF subtype diversity observed in clinical samples, enabling a longer‐term availability of CAF cell line models to support target discovery research. Our work also only included results from CAF coculturing with a single PDAC cell line, BxPC3. While we highlight sample‐to‐sample variability in CAF phenotypes corresponding to pathological differences across donors, our Perturb‐seq analysis was only performed on one immortalized cell line. Additionally, the scope of our work did not include functional characterization of CAF subtypes following perturbations. Finally, while we discussed several implications of the applicability of our work on illuminating the antitumor or protumor effects of CAF subtypes (e.g., specific CAF subtypes having significant correlation with immuno‐resistance, perturbing *AEBP1* transforming CAF phenotypes from a protumorigenic glycolytic state to an oxidative phosphorylation state), we acknowledge the necessity for further investigation as an important area for future work.

## 5 Conclusions

Our results demonstrated the utility of *in vitro* CAF cell lines and single‐cell perturbation assays for facilitating stromal target discovery efforts. Importantly, the heterogeneity of patient‐derived CAF cell lines could vary greatly, and profiling the heterogeneity of CAF cell lines could help highlight possible biases and inform cell line model selection in a hypothesis‐driven manner.

## Conflict of interest

ES, SKV, LL, HS, KN, AM, SN, FP, AMT, XY, and AKO are employees of Merck Sharp & Dohme LLC, a subsidiary of Merck & Co., Inc., Rahway, NJ, USA and may hold stock and/or options in Merck & Co., Inc., Rahway, NJ, USA.

## Author contributions

XY and AKO conceived the study; XY, AKO, FP, and AMT supervised the study; SKV, LL, HS, KN, AM, and SN performed experiments and produced the data; ES and SKV performed the computational preprocessing of the data; ES performed computational analysis, script writing, and visualization, and wrote the manuscript; XY, AKO, and SKV provided manuscript reviews.

## Supporting information


**Fig. S1.** Vector map of All‐in‐One pRDA_208 vector.
**Fig. S2.** Pre‐processing of *in vitro* CAF single‐cell RNA sequencing data.
**Fig. S3.** Pre‐processing of single‐cell RNA sequencing data of parental and hTERT‐immortalized BxPC3‐CAF co‐cultures.
**Fig. S4.** Characterization of CAF subtypes in untreated, BxPC3‐co‐cultured, and TGFβ1‐treated CAFs.
**Fig. S5.** Pre‐processing of BxPC3‐cocultured CAF Perturb‐seq data.
**Fig. S6.** Pre‐processing of CAF monoculture Perturb‐seq data.
**Fig. S7.** Detection of non‐perturbed cells in BxPC3‐co‐cultured CAF Perturb‐seq data with mixscape.
**Fig. S8.** Detection of non‐perturbed cells in CAF monoculture Perturb‐seq data with mixscape.
**Fig. S9.** Label transfer, clustering, and CAF subtype identification for BxPC3‐cocultured CAF Perturb‐seq data.
**Fig. S10.** Levels of IL6 and IL11 in BxPC3, CAF monocultures, and BxPC3‐co‐cultured CAF cell lines.
**Fig. S11.** Heterogeneity of untreated and TGFβ1‐treated CAFs.
**Fig. S12.** Pro‐tumorigenic effects of CAF cell lines on BxPC3 growth.
**Fig. S13.** Contractility of BxPC3, CAFs, and BxPC3‐CAF co‐cultures.
**Fig. S14.** hTERT‐immortalization of BxPC3‐co‐cultured CAF cell lines largely preserves the heterogeneity of parental lines.
**Fig. S15.** Single‐cell RNA sequencing highlights the context specificity of *in vitro* CAF activation.
**Fig. S16.** Differentially overexpressed genes from *in vitro* CAF activation.
**Fig. S17.** Expression of universal fibroblast markers across CAF subtypes in BxPC3‐CAF co‐cultures.
**Fig. S18.** Comparison between Perturb‐seq results from hTERT‐BxPC3‐donor‐1 and hTERT‐donor‐1 monoculture.
**Fig. S19.** Pathway enrichments of upregulated genes from CAF‐tumor co‐cultures using RCC, PANC10, and BxPC3 cell lines.
**Fig. S20.** Differentially expressed pan‐disease fibrosis genes after CAF‐tumor co‐culturing.
**Fig. S21.** Correlation between *in vitro* CAF subtypes in BxPC3‐CAF co‐cultures with primary CAF signatures from Cords et al. on primary breast cancer.

**Table S1.** Pathological information on CAF donors.
**Table S2.** Sequences of gRNAs used in the Perturb‐seq experiment.
**Table S3.** Upregulated and downregulated pathways from AEBP1 perturbation.
**Table S4.** Upregulated and downregulated pathways from SPATS2L perturbation.
**Table S5.** Upregulated and downregulated pathways from PTGS1 perturbation.
**Table S6.** Upregulated and downregulated pathways from TGFBR1 perturbation.

## Data Availability

The single‐cell RNA‐seq and Perturb‐seq data produced in this work are available at GEO with accession numbers GSE301398 and GSE306853, respectively.
