# Supplementary material for: In vitro models of cancer‐associated fibroblast heterogeneity uncover subtype‐specific effects of CRISPR perturbations
Source: Mol Oncol. 2025 Oct 27;20(5):1253–69. doi: 10.1002/1878-0261.70153 (PMC13155148; doi:10.1002/1878-0261.70153)
Supplement: Supplementary file 1 — Fig. S1. Vector map of All‐in‐One pRDA_208 vector. Fig. S2. Pre‐processing of in vitro CAF single‐cell RNA sequencing data. Fig. S3. Pre‐processing of single‐cell RNA sequencing data of parental and hTERT‐immortalized BxPC3‐CAF co‐cultures. Fig. S4. Characterization of CAF subtypes in untreated, BxPC3‐co‐cultured, and TGFβ1‐treated CAFs. Fig. S5. Pre‐processing of BxPC3‐cocultured CAF Perturb‐seq data. Fig. S6. Pre‐processing of CAF monoculture Perturb‐seq data. Fig. S7. Detection of non‐perturbed cells in BxPC3‐co‐cultured CAF Perturb‐seq data with mixscape. Fig. S8. Detection of non‐perturbed cells in CAF monoculture Perturb‐seq data with mixscape. Fig. S9. Label transfer, clustering, and CAF subtype identification for BxPC3‐cocultured CAF Perturb‐seq data. Fig. S10. Levels of IL6 and IL11 in BxPC3, CAF monocultures, and BxPC3‐co‐cultured CAF cell lines. Fig. S11. Heterogeneity of untreated and TGFβ1‐treated CAFs. Fig. S12. Pro‐tumorigenic effects of CAF cell lines on BxPC3 growth. Fig. S13. Contractility of BxPC3, CAFs, and BxPC3‐CAF co‐cultures. Fig. S14. hTERT‐immortalization of BxPC3‐co‐cultured CAF cell lines largely preserves the heterogeneity of parental lines. Fig. S15. Single‐cell RNA sequencing highlights the context specificity of in vitro CAF activation. Fig. S16. Differentially overexpressed genes from in vitro CAF activation. Fig. S17. Expression of universal fibroblast markers across CAF subtypes in BxPC3‐CAF co‐cultures. Fig. S18. Comparison between Perturb‐seq results from hTERT‐BxPC3‐donor‐1 and hTERT‐donor‐1 monoculture. Fig. S19. Pathway enrichments of upregulated genes from CAF‐tumor co‐cultures using RCC, PANC10, and BxPC3 cell lines. Fig. S20. Differentially expressed pan‐disease fibrosis genes after CAF‐tumor co‐culturing. Fig. S21. Correlation between in vitro CAF subtypes in BxPC3‐CAF co‐cultures with primary CAF signatures from Cords et al. on primary breast cancer. [file MOL2-20-1253-s002.pdf]

Supplementary Figures

S1. Vector map of All-in-One pRDA_208 vector 2

S2. Pre-processing of in vitro CAF single-cell RNA sequencing data 3

S3. Pre-processing of single-cell RNA sequencing data of parental and hTERT-immortalized BxPC3-CAF co-cultures 4

S4. Characterization of CAF subtypes in untreated, BxPC3-co-cultured, and TGFβ1-treated CAFs ... 5

S5. Pre-processing of BxPC3-cocultured CAF Perturb-seq data 6

S6. Pre-processing of CAF monoculture Perturb-seq data 7

S7. Detection of non-perturbed cells in BxPC3-co-cultured CAF Perturb-seq data with mixscape 8

S8. Detection of non-perturbed cells in CAF monoculture Perturb-seq data with mixscape 9

S9. Label transfer, clustering, and CAF subtype identification for BxPC3-cocultured CAF Perturb-seq data 10

S10. Levels of IL6 and IL11 in BxPC3, CAF monocultures, and BxPC3-co-cultured CAF cell lines .. 11

S11. Heterogeneity of untreated and TGFβ1-treated CAFs 12

S12. Pro-tumorigenic effects of CAF cell lines on BxPC3 growth 13

S13. Contractility of BxPC3, CAFs, and BxPC3-CAF co-cultures 14

S14. hTERT-immortalization of BxPC3-co-cultured CAF cell lines largely preserves the heterogeneity of parental lines 15

S15. Single-cell RNA sequencing highlights the context specificity of in vitro CAF activation 16

S16. Differentially overexpressed genes from in vitro CAF activation 17

S17. Expression of universal fibroblast markers across CAF subtypes in BxPC3-CAF co-cultures ... 18

S18. Comparison between Perturb-seq results from hTERT-BxPC3-donor-1 and hTERT-donor-1 monoculture 18

S19. Pathway enrichments of upregulated genes from CAF-tumor co-cultures using RCC, PANC10, and BxPC3 cell lines 19

S20. Differentially expressed pan-disease fibrosis genes after CAF-tumor co-culturing 19

S21. Correlation between in vitro CAF subtypes in BxPC3-CAF co-cultures with primary CAF signatures from Cords et al. on primary breast cancer 20

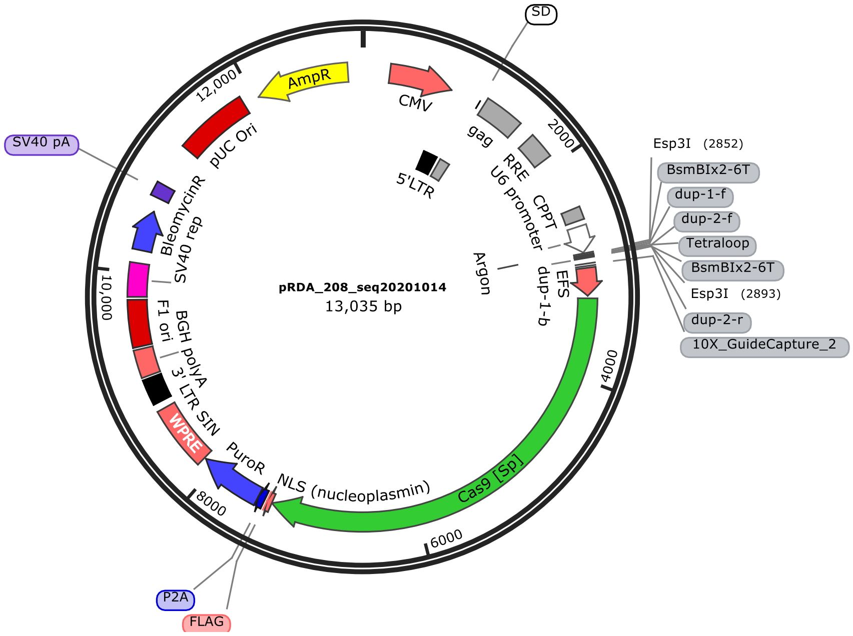

Figure S1. Vector map of All-in-One pRDA_208 vector.

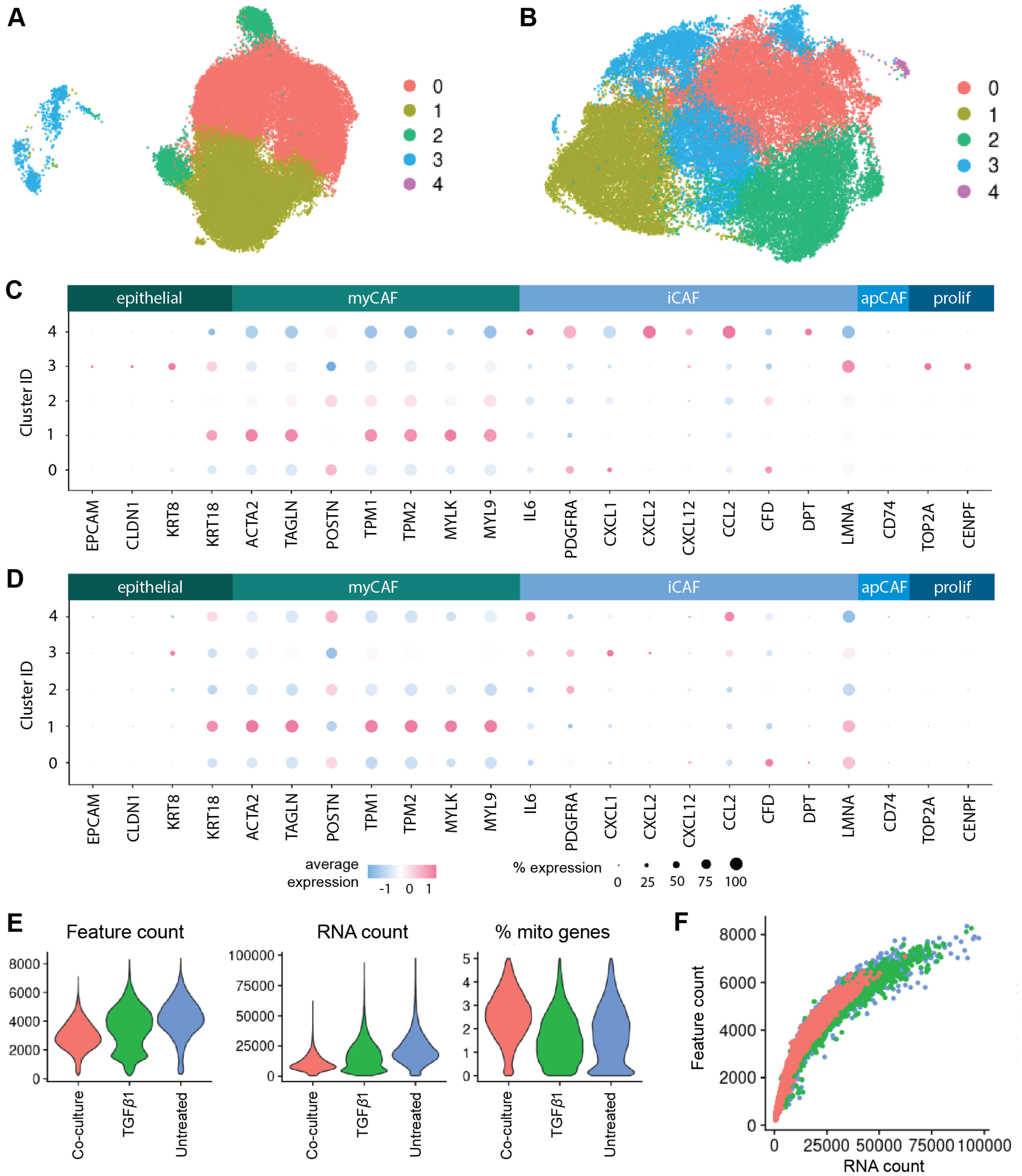

Figure S2. Pre-processing of *in vitro* CAF single-cell RNA sequencing data. (**A**) UMAP visualization of scRNA-seq data of the pooled untreated, TGFβ1-treated, and BxPC3-co-cultured CAF samples before removal of epithelial cells (cluster 3), and (**B**) the same for after removal of epithelial cells. (**C**) Dot plot showing expression of markers of epithelial cells, myofibroblastic CAFs, inflammatory CAFs, antigen-presenting CAFs, and proliferation before removal of epithelial cells, and (**D**) the same for after removal of epithelial cells. (**E**) Violin plots and (**F**) scatter plot for quality check.

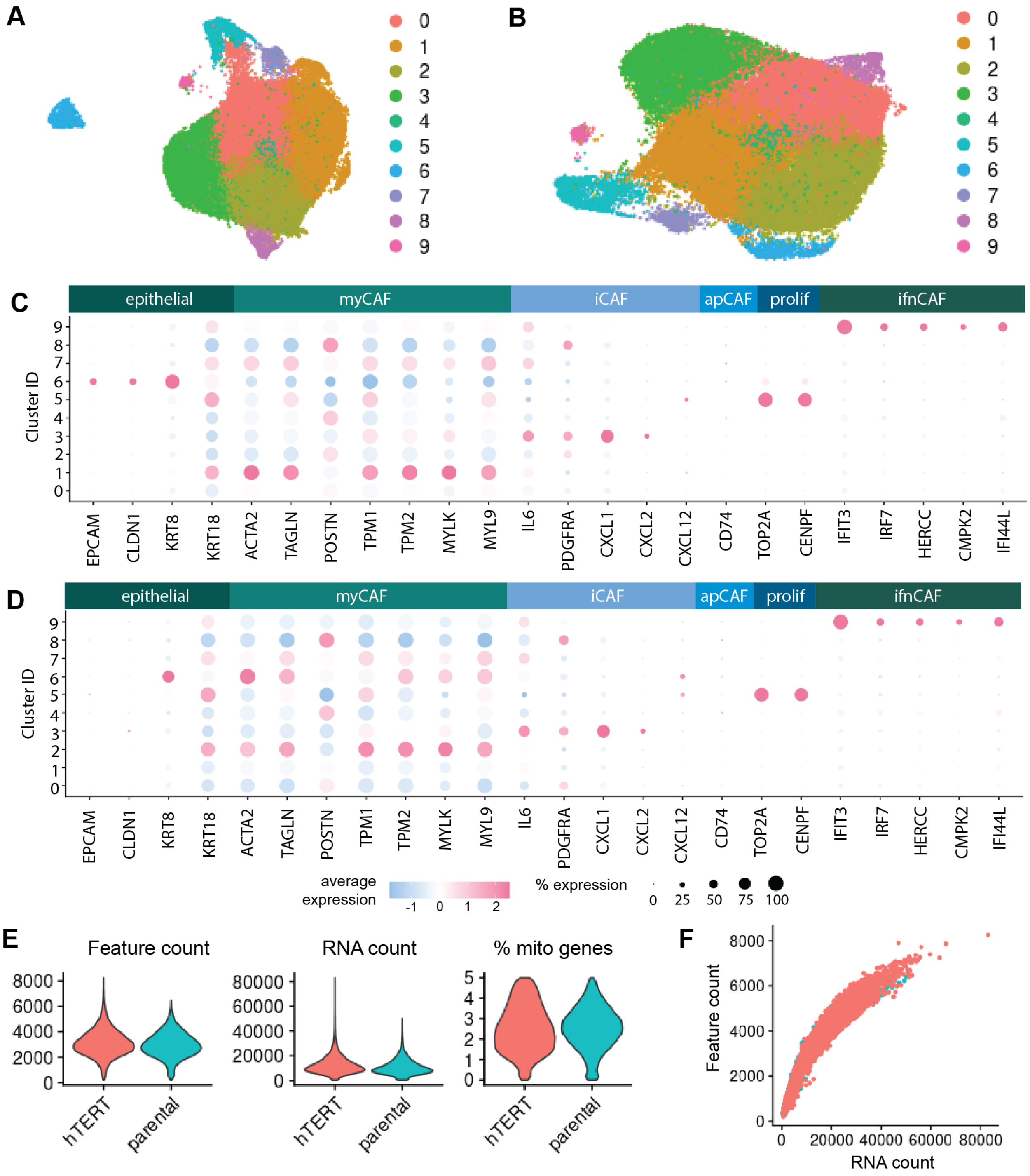

Figure S3. Pre-processing of single-cell RNA sequencing data of parental and hTERT-immortalized BxPC3-CAF co-cultures. (**A**) UMAP visualization of scRNA-seq data of the pooled parental and hTERT-immortalized BxPC3-CAF co-cultures before removal of epithelial cells (cluster 6), and (**B**) the same for after removal of epithelial cells. (**C**) Dot plot showing expression of markers of epithelial cells, myofibroblastic CAFs, inflammatory CAFs, antigen-presenting CAFs, interferon CAFs, and proliferation before removal of epithelial cells, and (**D**) the same for after removal of epithelial cells. (**E**) Violin plots and (**F**) scatter plot for quality check.

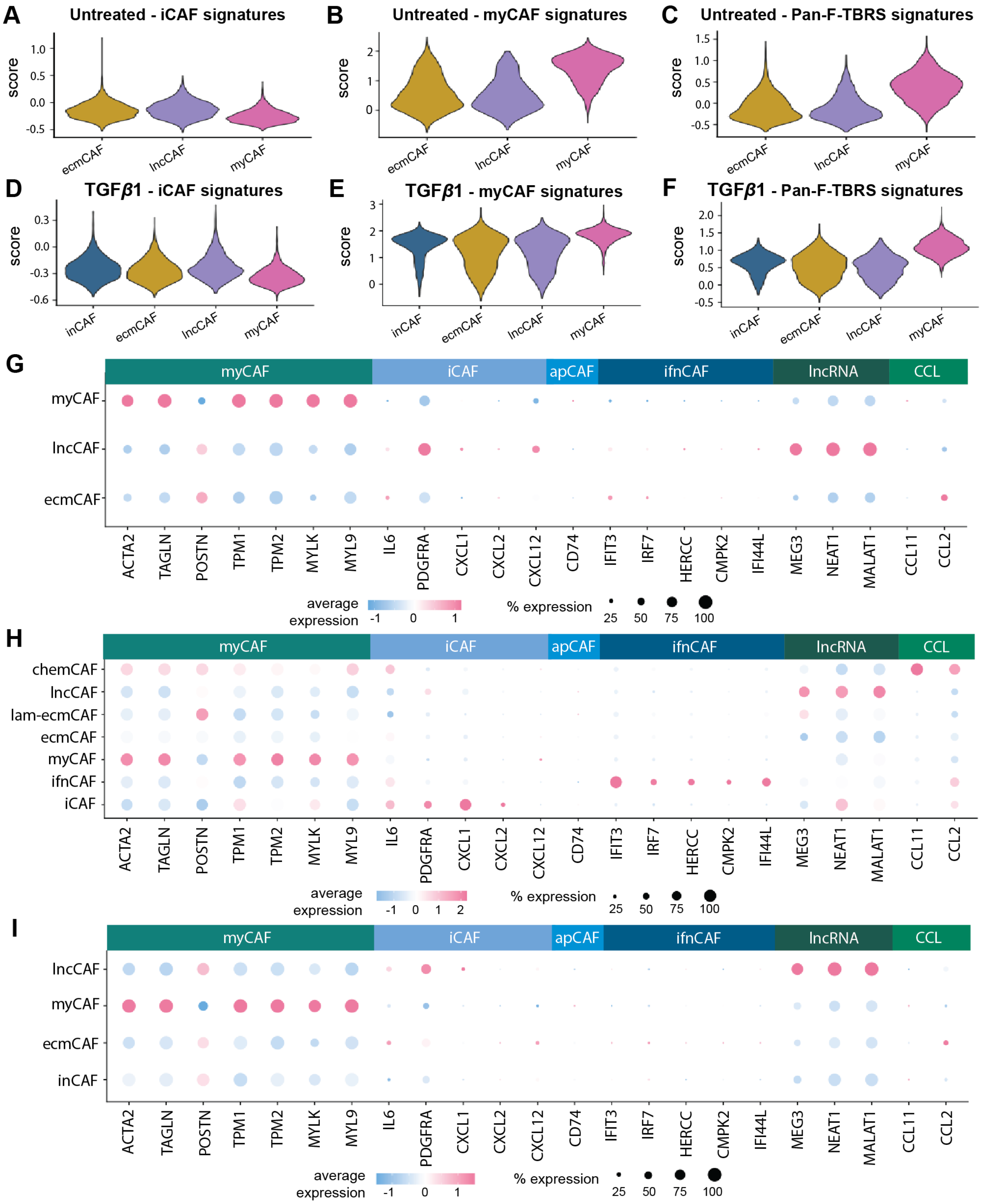

Figure S4. Characterization of CAF subtypes in untreated, BxPC3-co-cultured, and TGFβ1-treated CAFs. (**A**) Scores of primary iCAF signatures annotated by Elyada et al. across subtypes in untreated CAF samples, and the same plots for (**B**) primary myCAF signatures by Elyada et al. and (**C**) pan-fibroblast TGF-β response signature by Mariathasan et al. (**D, E, F**) The same plots for TGFβ1-treated CAFs. (**G**) Dot plot showing expression of markers of CAF subtypes in untreated CAFs, (**H**) BxPC3-co-cultured CAFs, and (**I**) TGFβ1-treated CAFs.

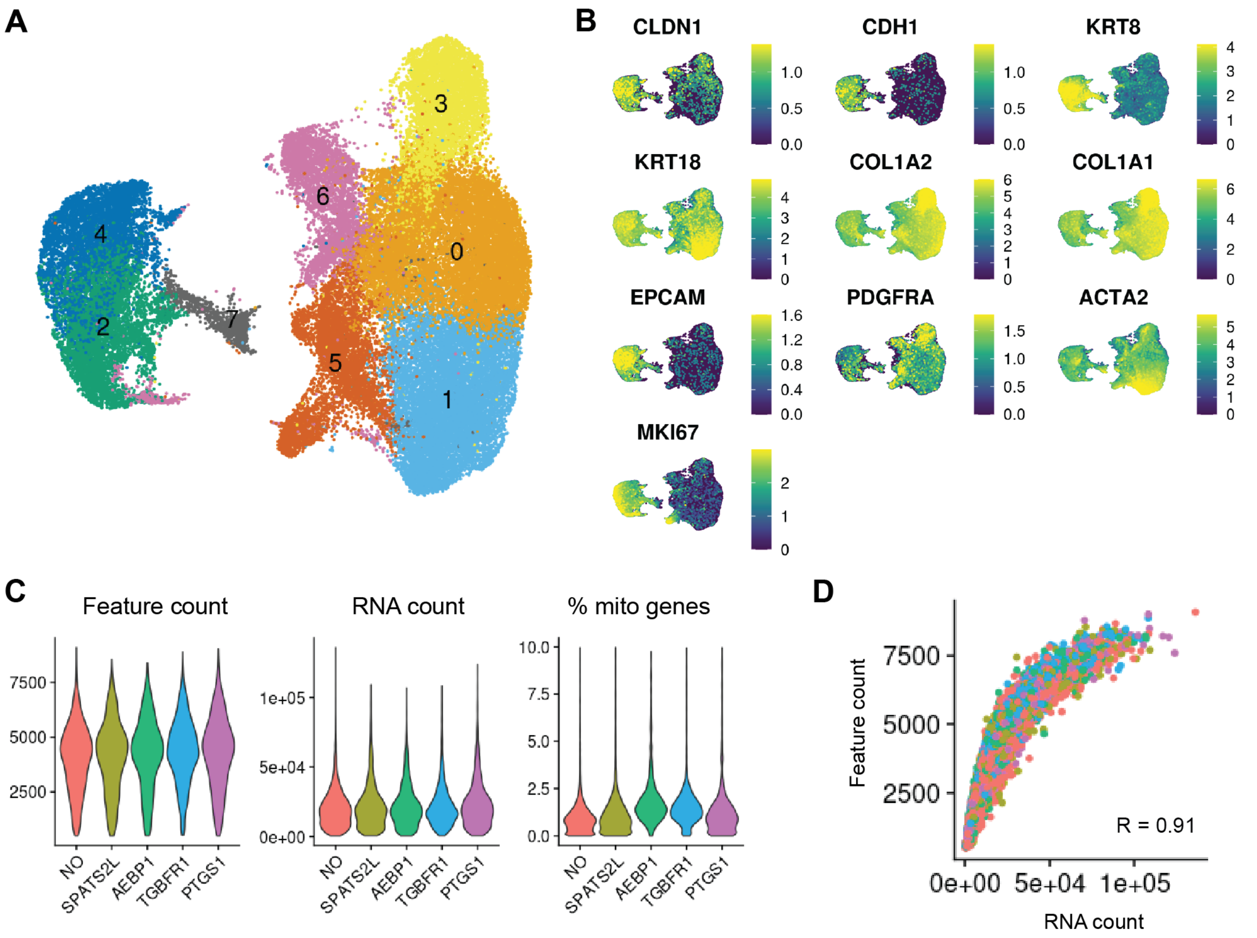

Figure S5. Pre-processing of BxPC3-cocultured CAF Perturb-seq data. (**A**) UMAP visualization of clusters in the co-culture Perturb-seq data. Clusters 2,4, and 7 are removed in downstream analysis due to expression of epithelial markers *CLDN1*, *CDH1*, *KRT8*, *KRT18*, and *EPCAM*. (**B**) Expression of epithelial, CAF, and proliferation markers. (**C**) Violin plots and (**D**) scatter plot for quality check.

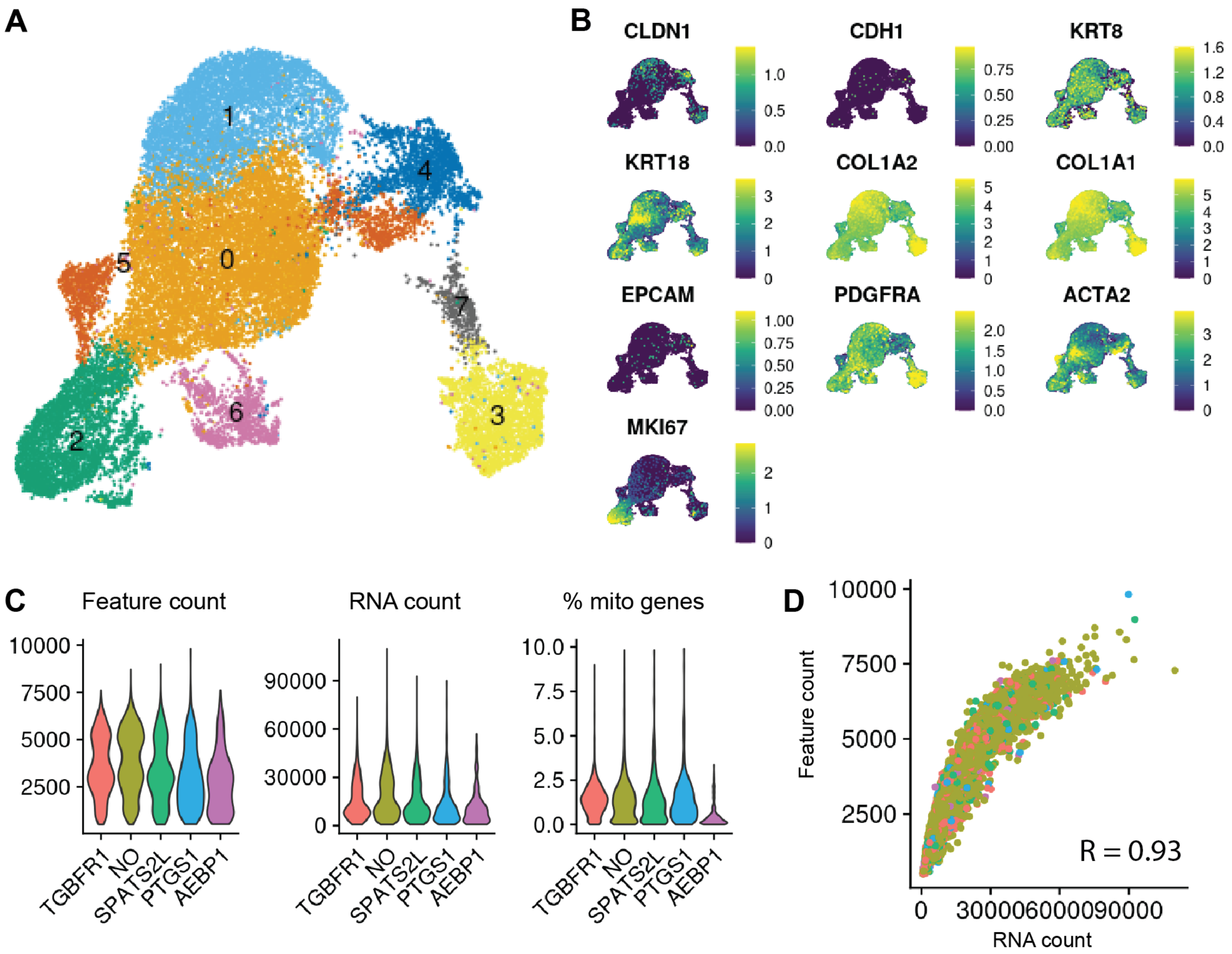

Figure S6. Pre-processing of CAF monoculture Perturb-seq data. (**A**) UMAP visualization of clusters in the monoculture Perturb-seq data. No cluster distinctively expresses all epithelial markers *CLDN1*, *CDH1*, *KRT8*, *KRT18*, and *EPCAM*. (**B**) Expression of epithelial, CAF, and proliferation markers. (**C**) Violin plots and (**D**) scatter plot for quality check.

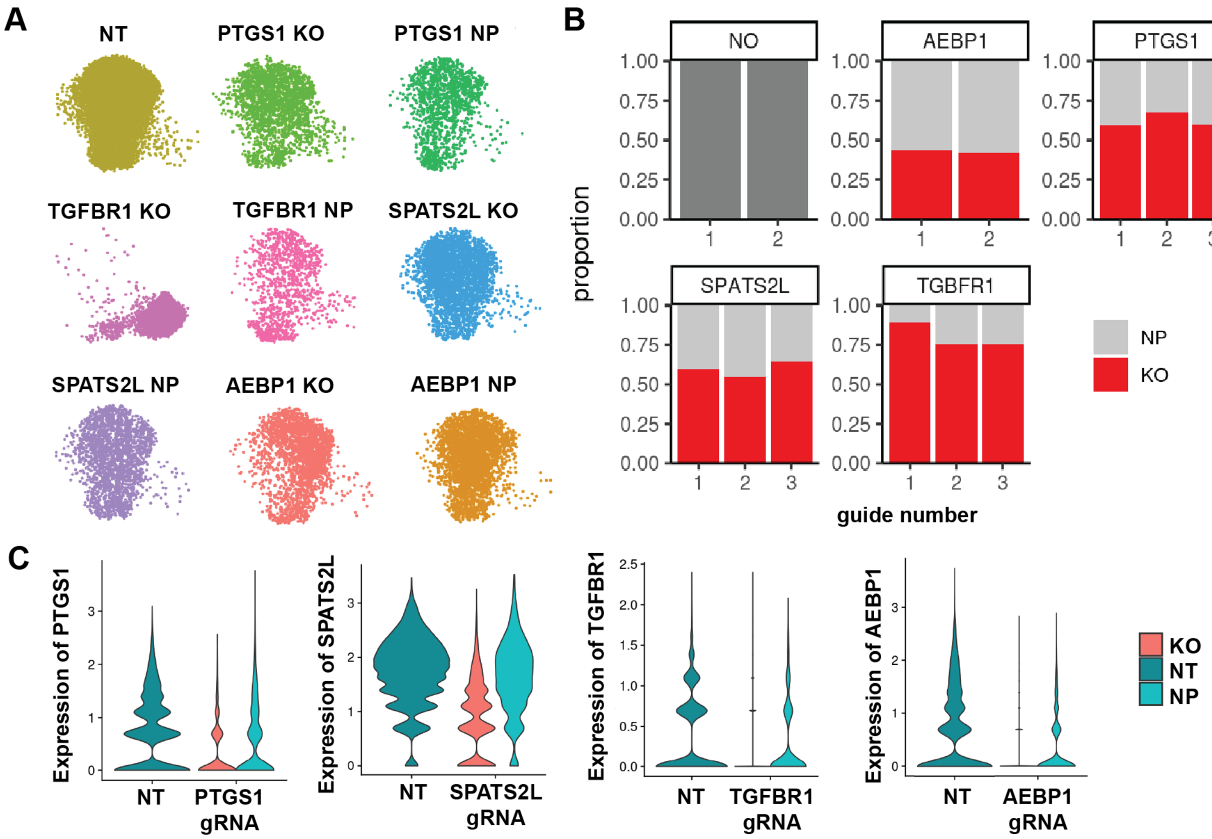

Figure S7. Detection of non-perturbed cells in BxPC3-co-cultured CAF Perturb-seq data with mixscape. (**A**) UMAP visualization of perturbation scores, split according to classes predicted by mixscape (NT = non-transfected, KO = knock-out, NP = non-perturbed). (**B**) Perturbation efficiency per mixscape class. (**C**) Expression of *PTGS1*, *SPATS2L*, *TGFBR1*, and *AEBP1* per mixscape class from the transfection or non-transfection with the corresponding gRNAs.

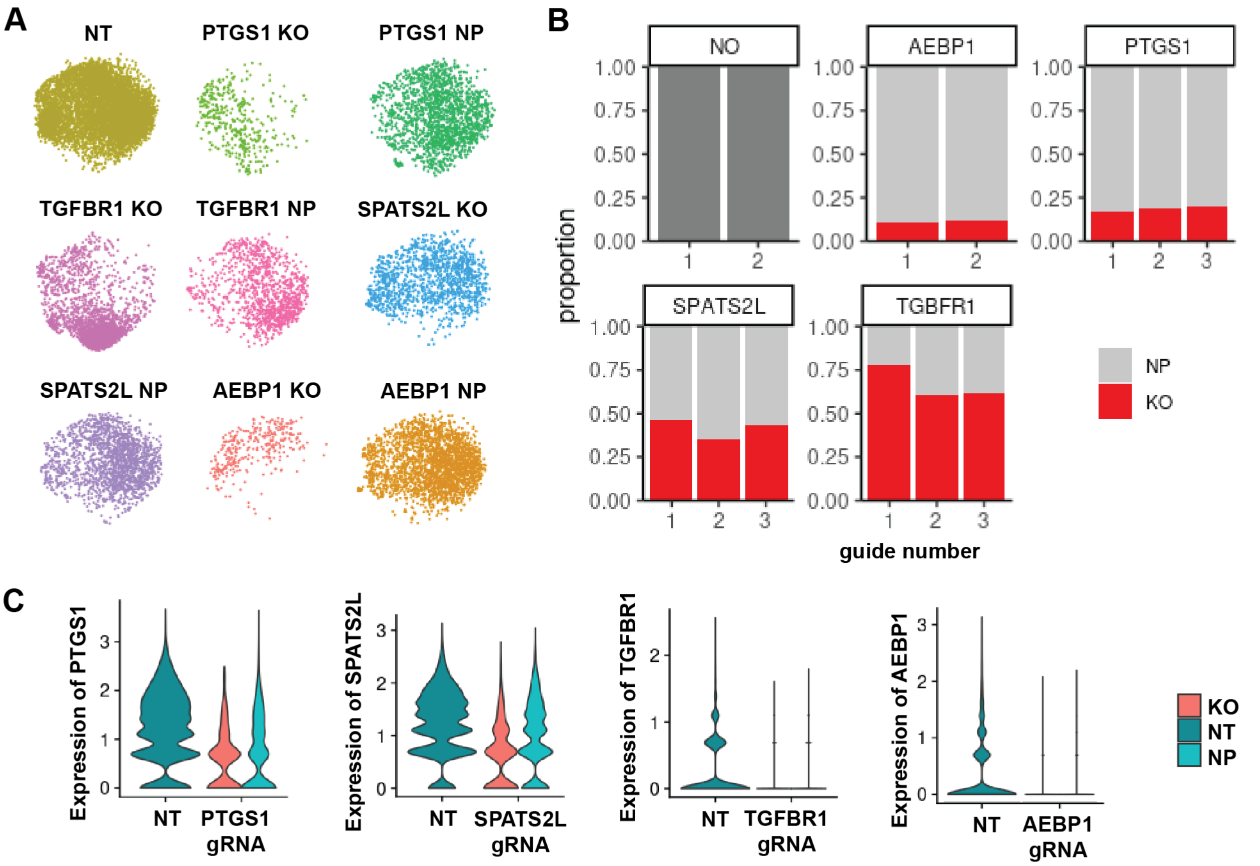

Figure S8. Detection of non-perturbed cells in CAF monoculture Perturb-seq data with mixscape. (**A**) UMAP visualization of perturbation scores, split according to classes predicted by mixscape (NT = non-transfected, KO = knock-out, NP = non-perturbed). (**B**) Perturbation efficiency per mixscape class. (**C**) Expression of *PTGS1*, *SPATS2L*, *TGFBR1*, and *AEBP1* per mixscape class from the transfection or non-transfection with the corresponding gRNAs.

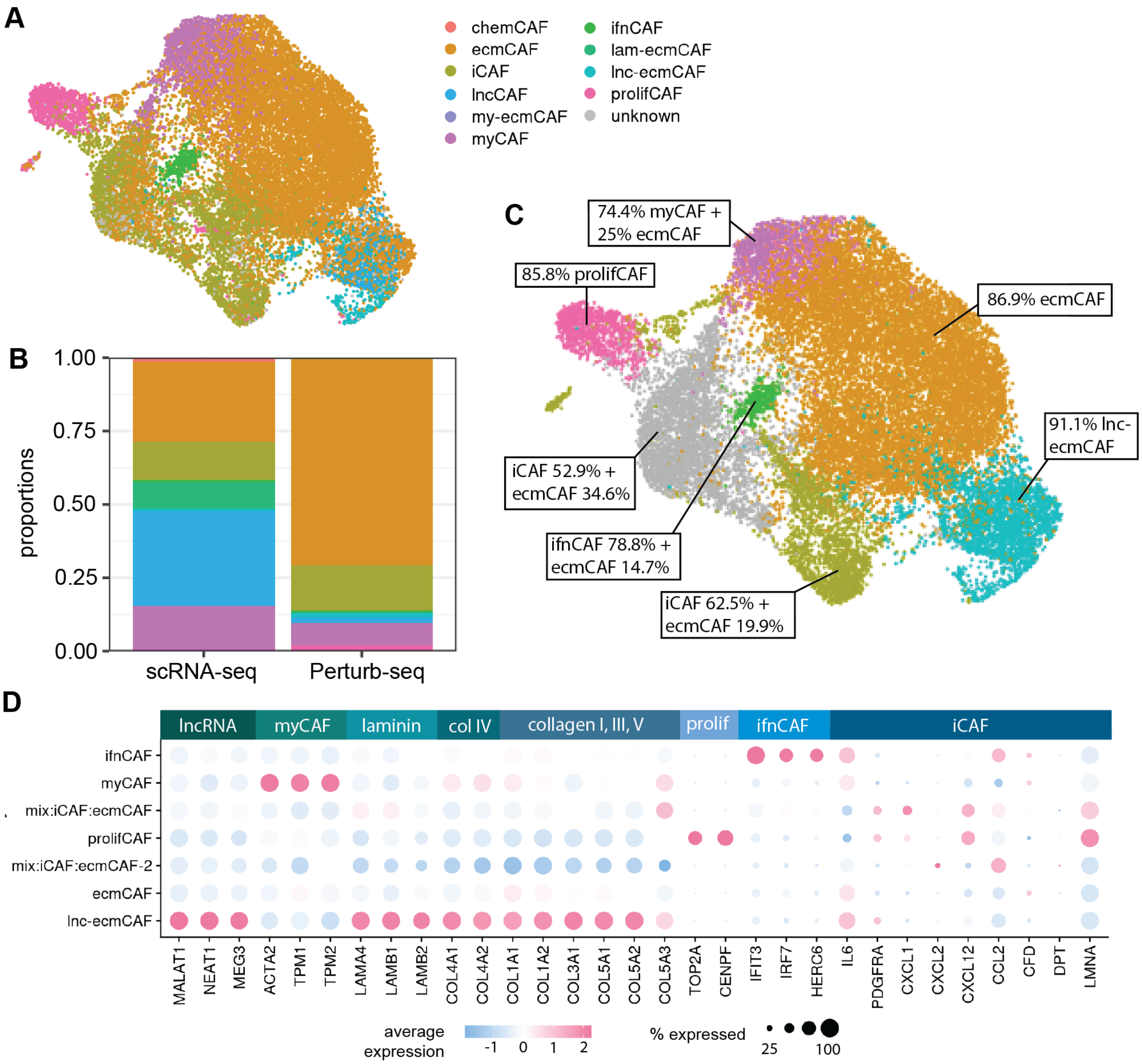

Figure S9. Label transfer, clustering, and CAF subtype identification for BxPC3-cocultured CAF Perturb-seq data. **(A)** Visualization of the Perturb-seq data with UMAP embeddings computed from Perturb-seq and cluster labels transferred from scRNA-seq. **(B)** Proportions of CAF subtypes in the hTERT-BxPC3-donor-1 scRNA-seq sample and the non-transfected (control) hTERT-BxPC3-donor-1 Perturb-seq sample. **(C)** Clustering results of the Perturb-seq data, with annotations of major subtype compositions. Only subtypes that make up > 10% of each cluster are annotated. **(D)** Dot plot showing expression of markers of extracellular matrix, myCAFs, iCAFs, ifnCAFs, proliferation, and long non-coding RNAs.

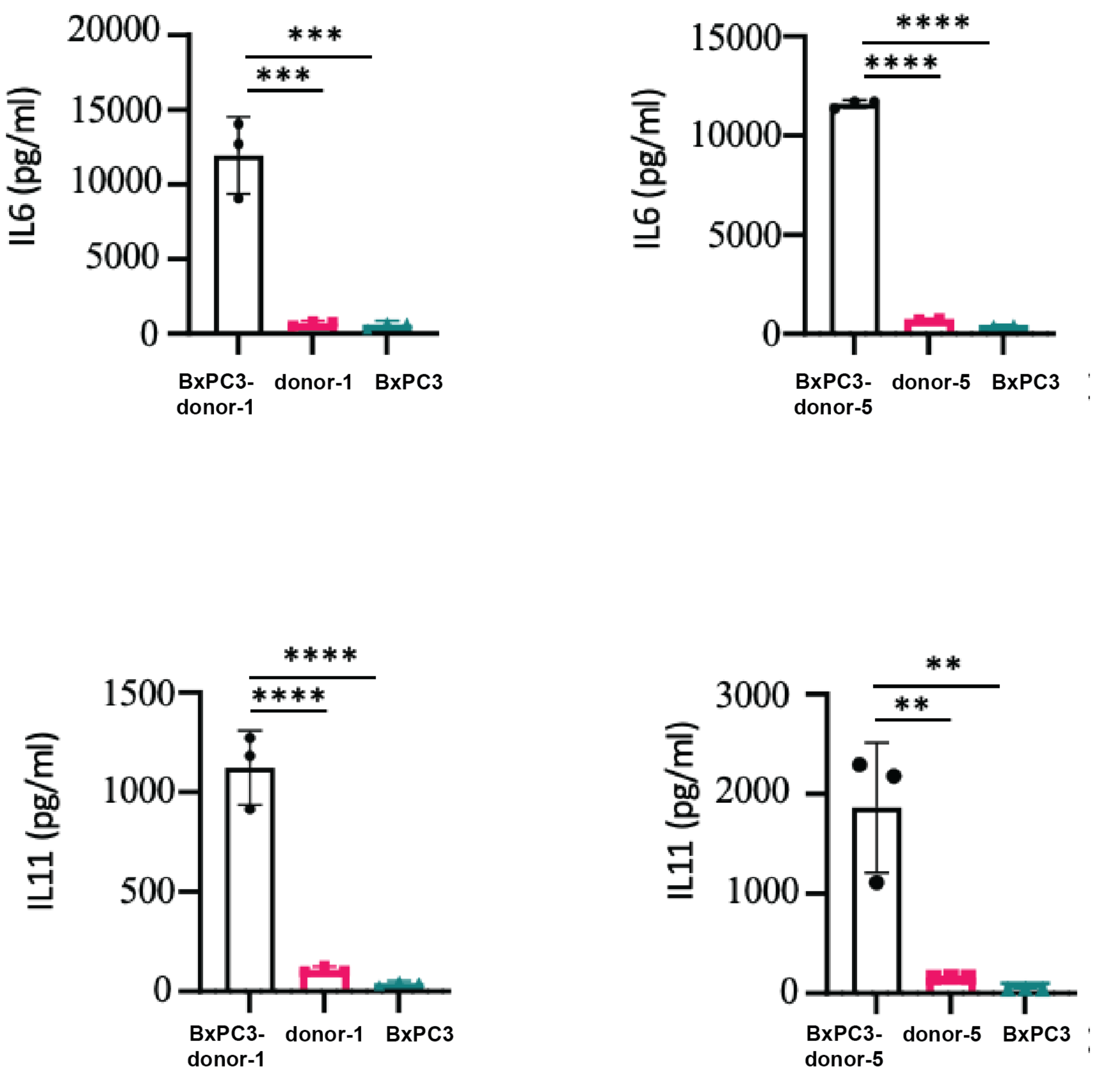

Figure S10. Levels of IL6 and IL11 in BxPC3, CAF monocultures, and BxPC3-co-cultured CAF cell lines. IL6 was measured after 24 hours, whereas IL11 was measured after 72 hours.

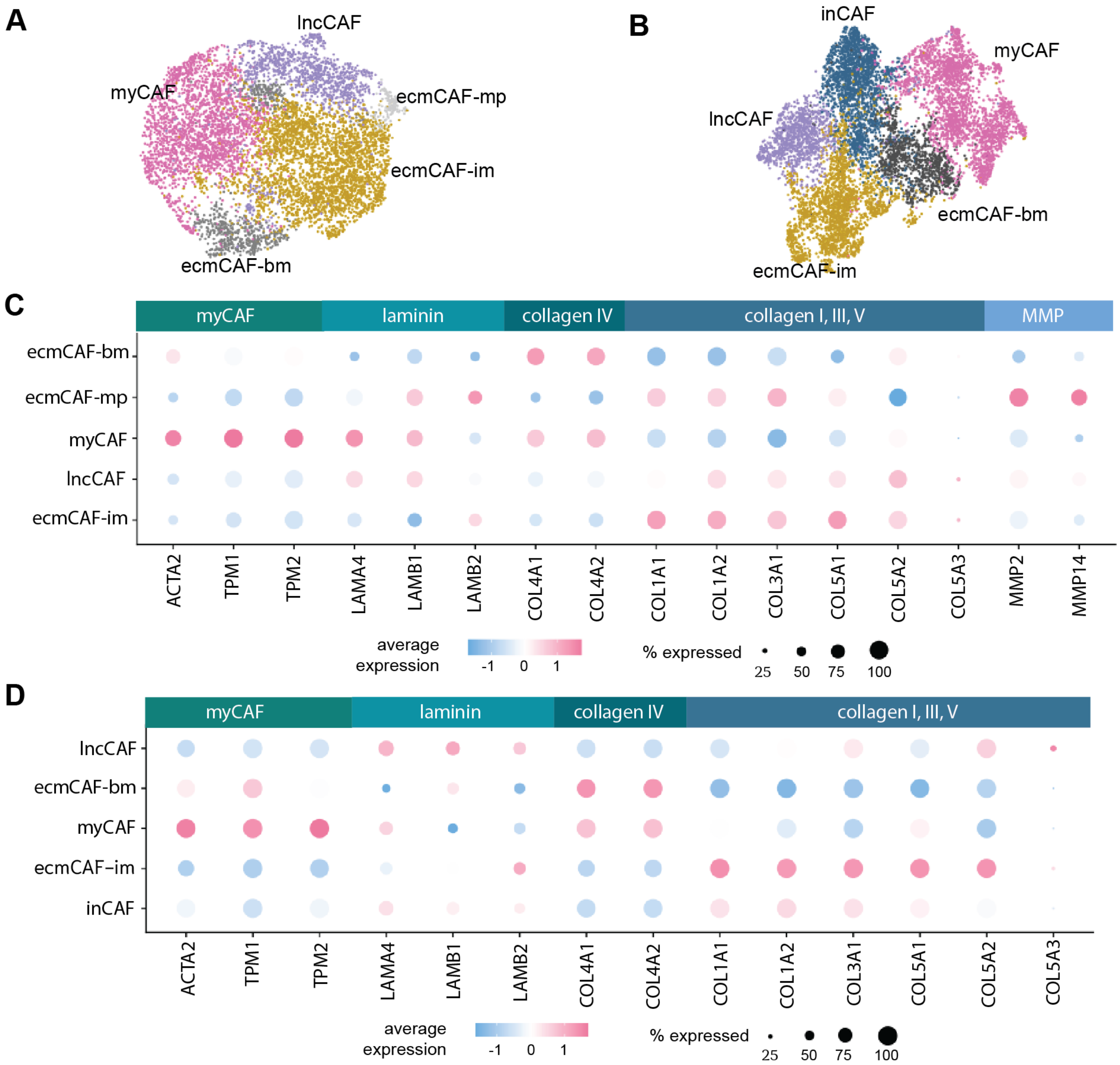

Figure S11. Heterogeneity of untreated and TGFβ1-treated CAFs. (**A**) UMAP visualization of scRNA-seq data of the untreated CAF samples and (**B**) TGFβ1-treated CAFs. (**C**) Dot plot showing expression of markers of extracellular matrix and myCAFs in untreated CAFs and (**D**) TGFβ1-treated CAFs.

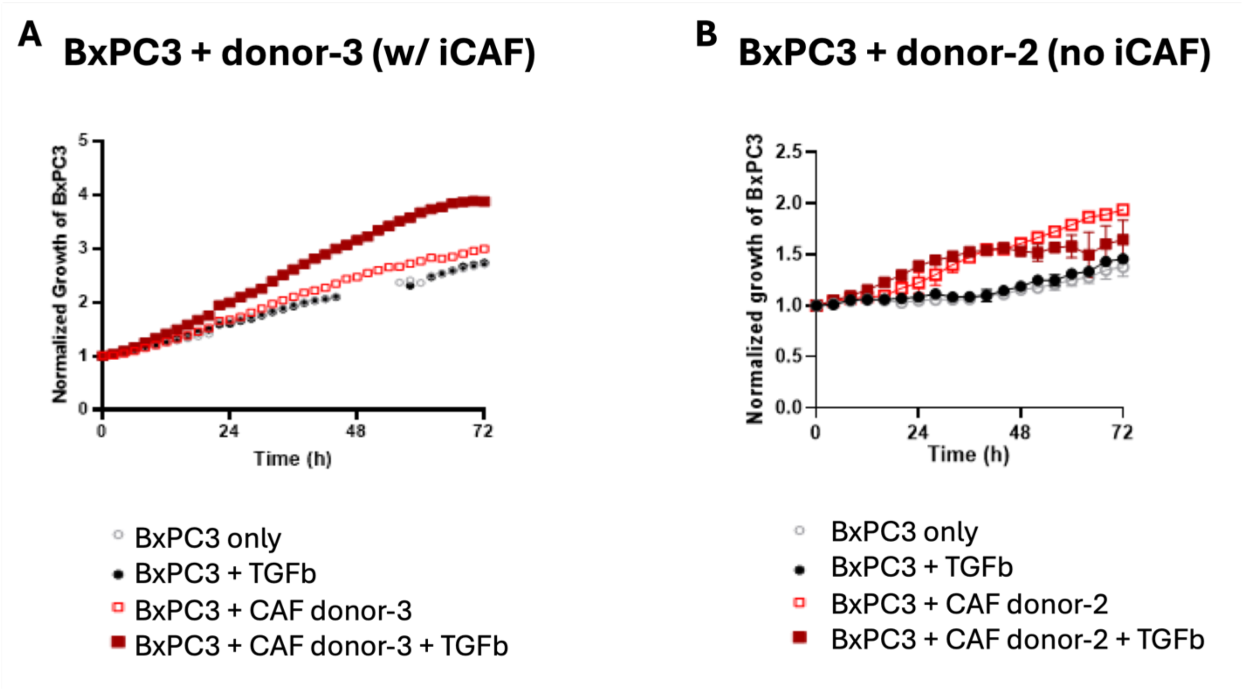

Figure S12. Pro-tumorigenic effects of CAF cell lines on BxPC3 growth. (**A**) The growth of BxPC3 arising from BxPC3 mono-culture, TGFβ treatment, BxPC3-CAF co-culture, and BxPC3-CAF co-culture + TGFβ treatment for iCAF-rich donor-3 CAFs and (**B**) the same for iCAF-deficient donor-2 CAFs.

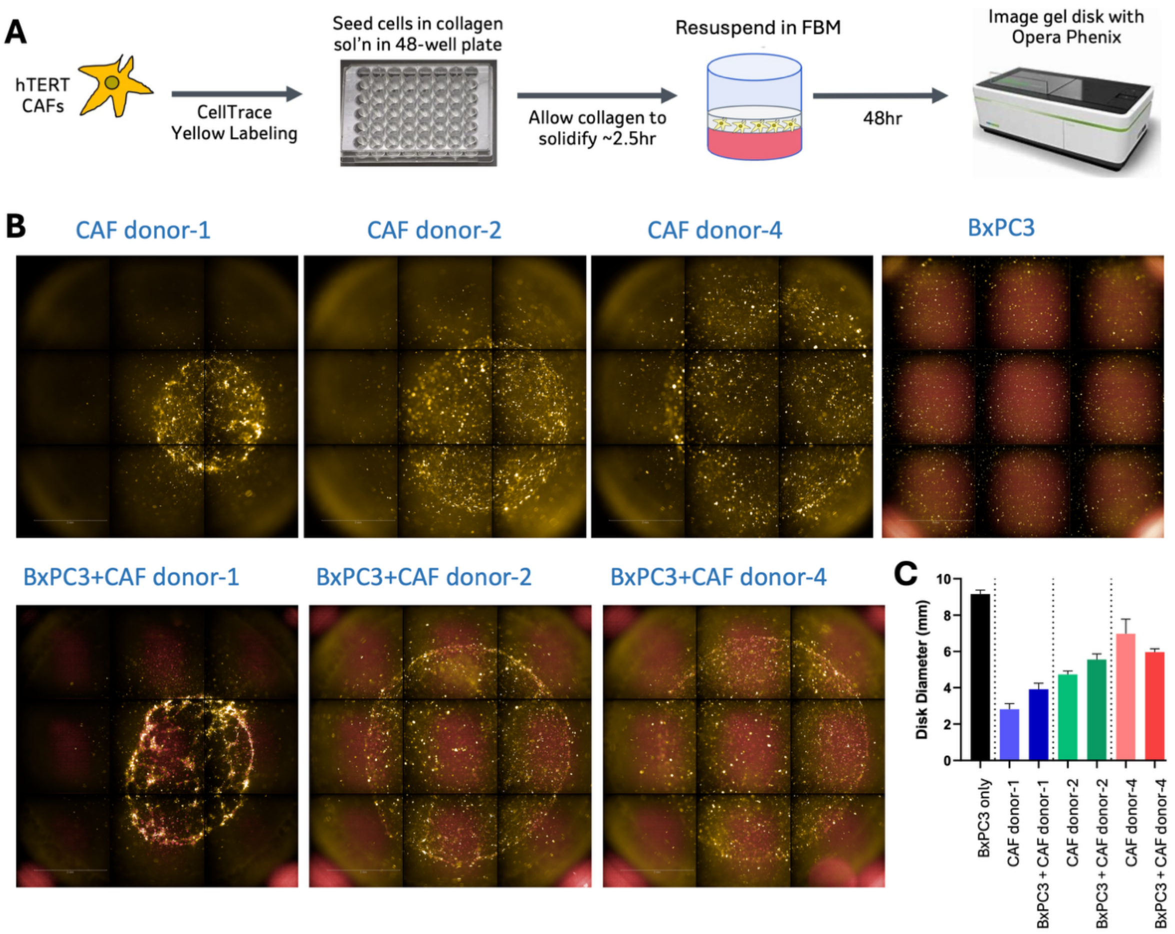

Figure S13. Contractility of BxPC3, CAFs, and BxPC3-CAF co-cultures. (A) Schematics of the contractility assay. (B) Contractility was determined by measuring the diameter of gel disk from multiple point and averaging the lengths. (C) Contractility measurements of BxPC3, CAFs, and BxPC3-CAF co-cultures.

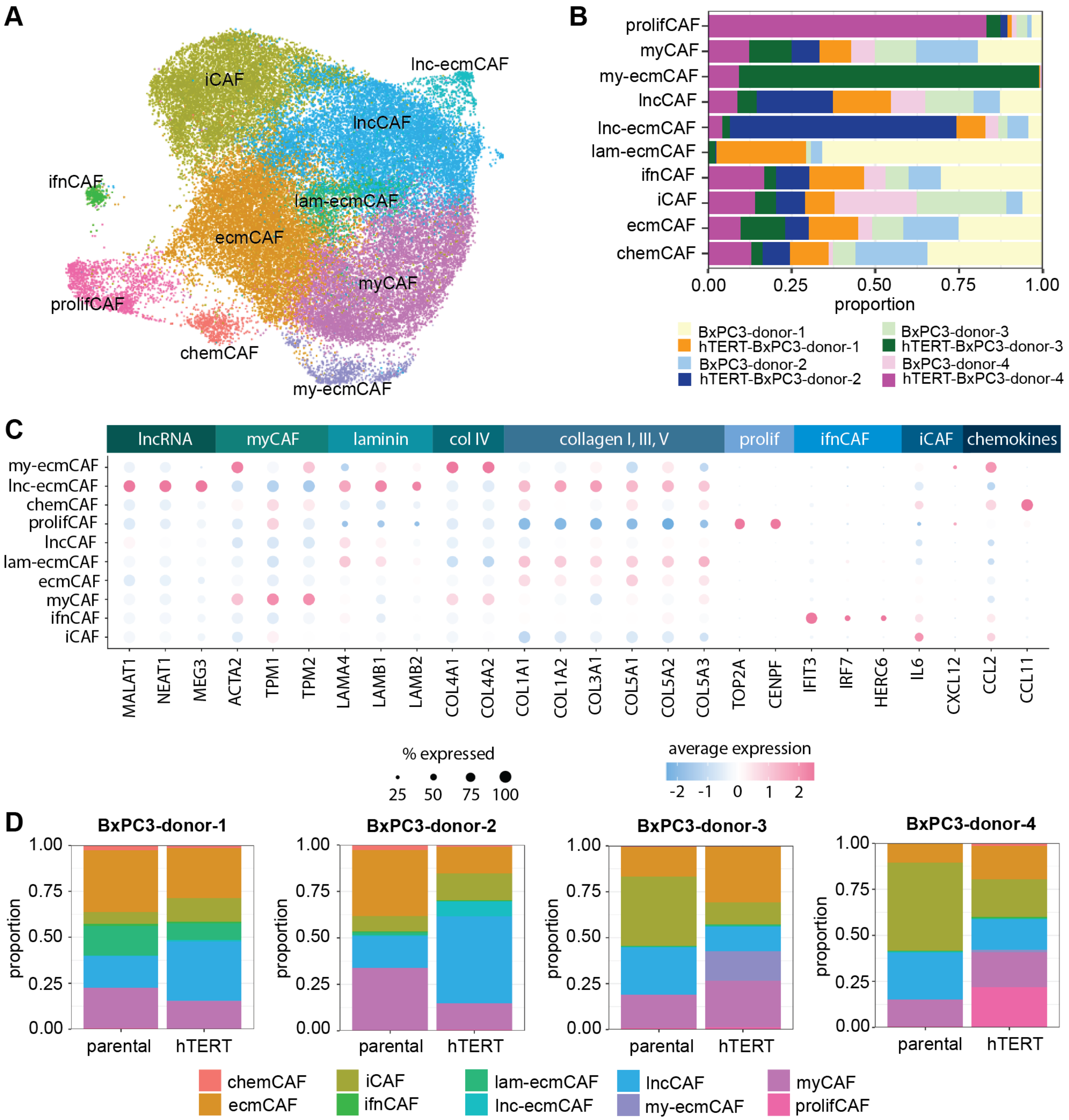

Figure S14. hTERT-immortalization of BxPC3-co-cultured CAF cell lines largely preserves the heterogeneity of parental lines. **(A)** UMAP visualization of parental and hTERT-immortalized BxPC3-CAF co-cultures, labeled with identified CAF subtypes. **(B)** Distribution of samples across different subtypes. **(C)** Dot plot showing expression of markers of extracellular matrix, myofibroblastic CAFs, inflammatory CAFs, interferon CAFs, proliferation, chemokines, and long non-coding RNAs. **(D)** Subtype proportions of parental versus hTERT-immortalized BxPC3-CAF co-cultures.

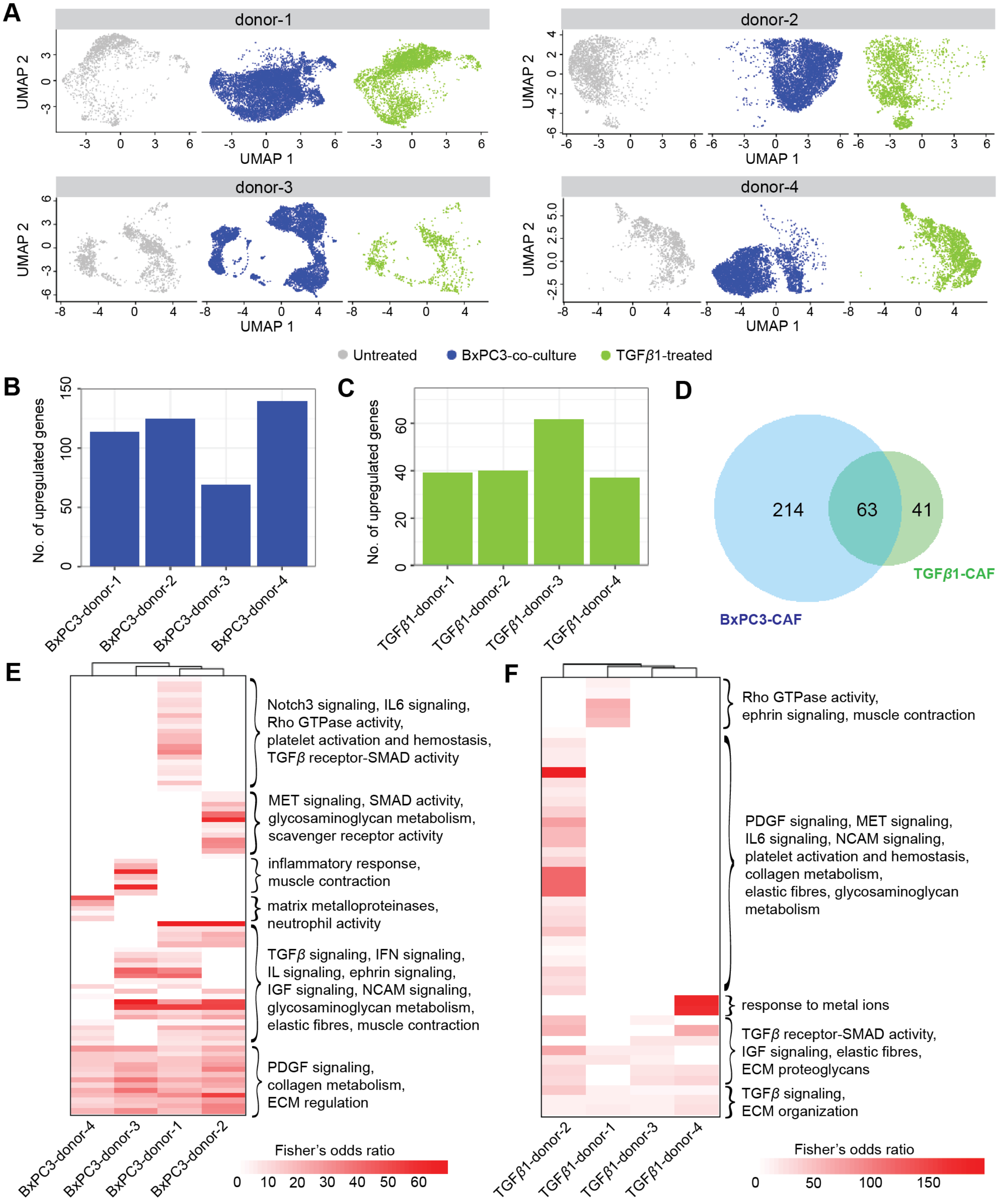

Figure S15. Single-cell RNA sequencing highlights the context specificity of *in vitro* CAF activation. (**A**) UMAP visualizations of untreated, BxPC3-co-cultured, and TGFβ1-treated CAFs across four samples. (**B**) Number of differentially overexpressed genes in BxPC3-co-cultured CAFs relative to untreated CAFs, and (**C**) the same for TGFβ1-treated CAFs (Benjamini-Hochberg adjusted p-value ≤ 0.01, average log2 fold-change ≥ 1). (**D**) Venn diagram showing the number of differentially overexpressed genes uniquely or commonly found from BxPC3 co-culturing or TGFβ1 treatment. (**E**)

Heatmap showing the significantly enriched Reactome pathways from BxPC3 co-culturing, and (**F**) the same for TGFβ1 treatment (Benjamini-Hochberg adjusted p-value $\leq$ 0.01).

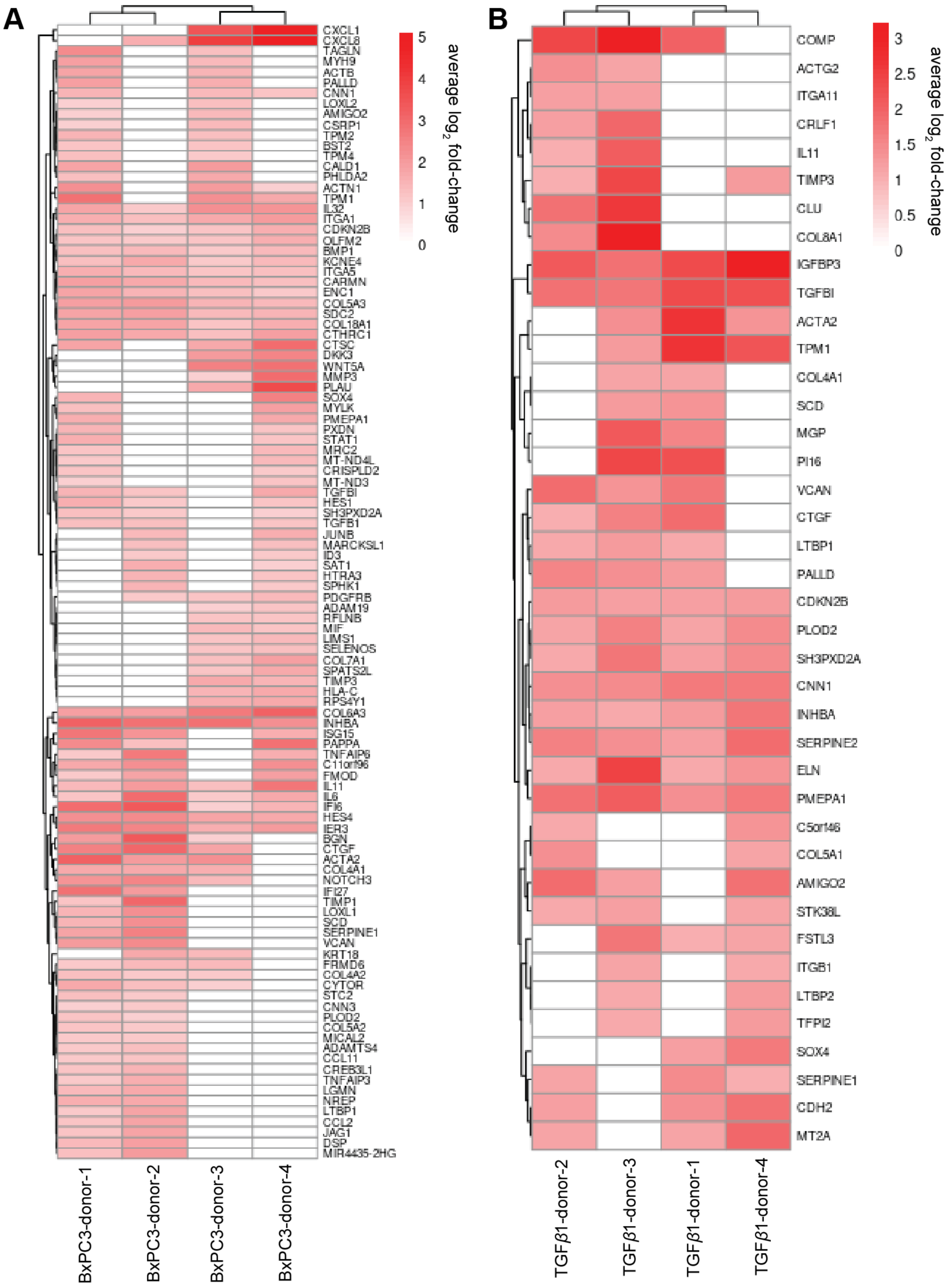

Figure S16. Differentially overexpressed genes from *in vitro* CAF activation. (**A**) Heatmap showing the genes that are significantly overexpressed in at least two samples from BxPC3-CAF co-culturing relative to untreated CAFs, and (**B**) the same for TGFβ1-treated CAFs relative to untreated CAFs (Benjamini-Hochberg adjusted p-value $\leq$ 0.01, average log2 fold-change $\geq$ 1).

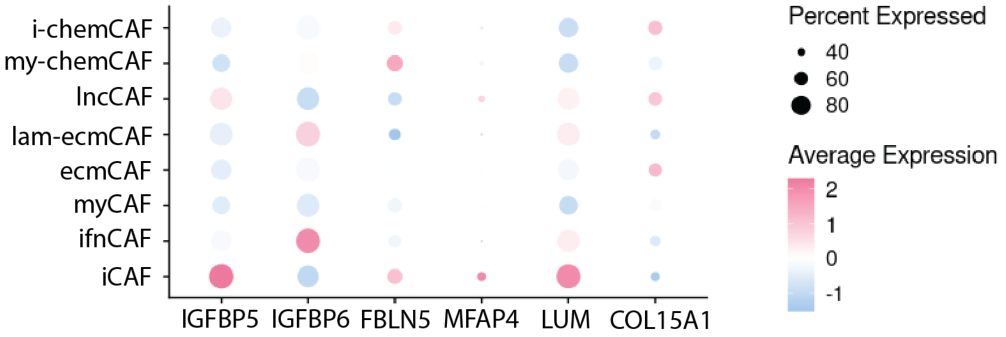

Figure S17. Expression of universal fibroblast markers across CAF subtypes in BxPC3-CAF co-cultures.

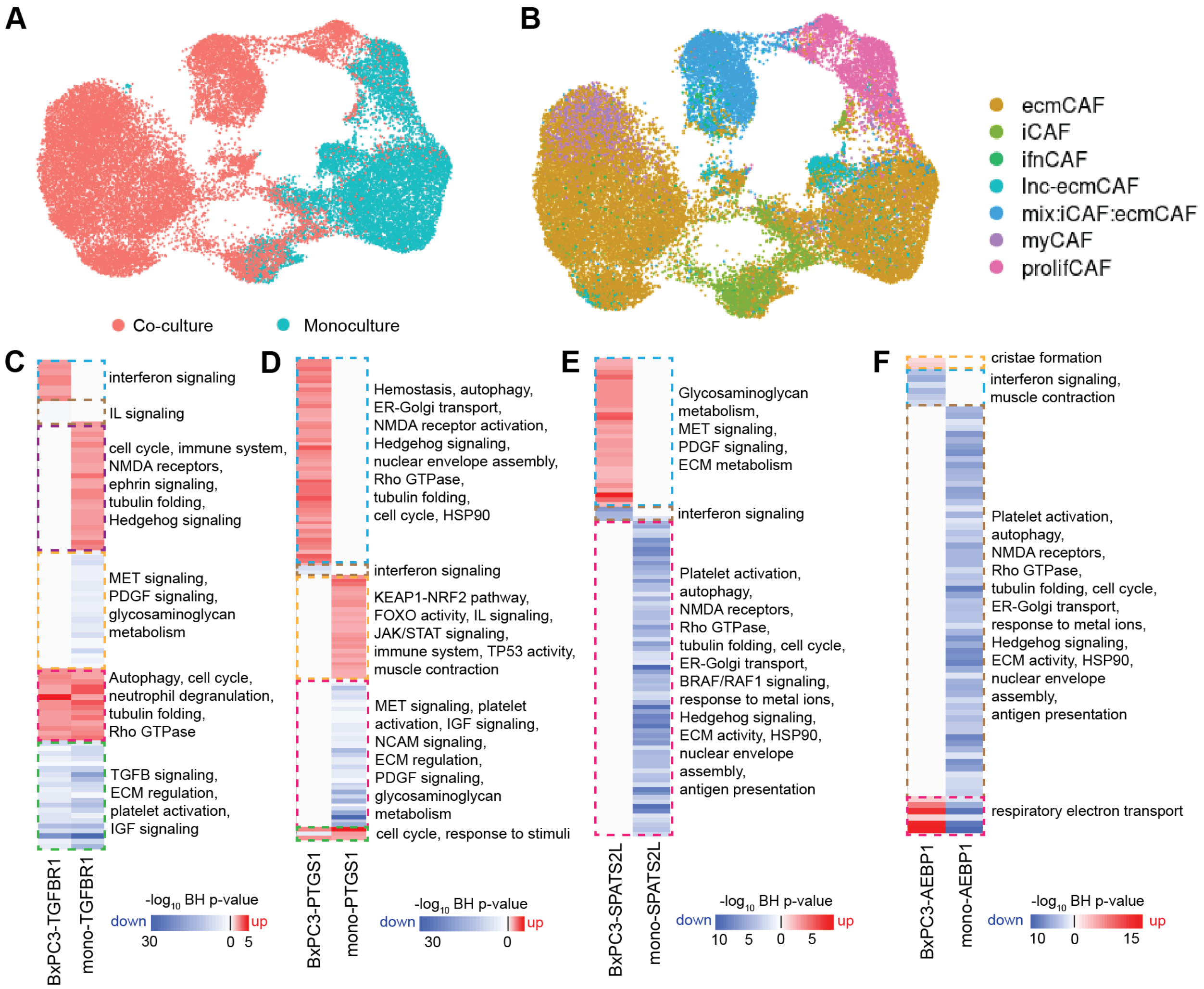

Figure S18. Comparison between Perturb-seq results from hTERT-BxPC3-donor-1 and hTERT-donor-1 monoculture. (**A**) UMAP visualization of all the cells in the Perturb-seq experiments performed on both cell lines. (**B**) UMAP visualization labelled according to CAF subtypes. (**C**) Reactome pathways that are significantly enriched for differentially expressed genes from *TGFBR1*

perturbation performed on both cell lines, and **(D)** the same for *PTGS1*, **(E)** *SPATS2L*, and **(F)** *AEBP1* perturbations.

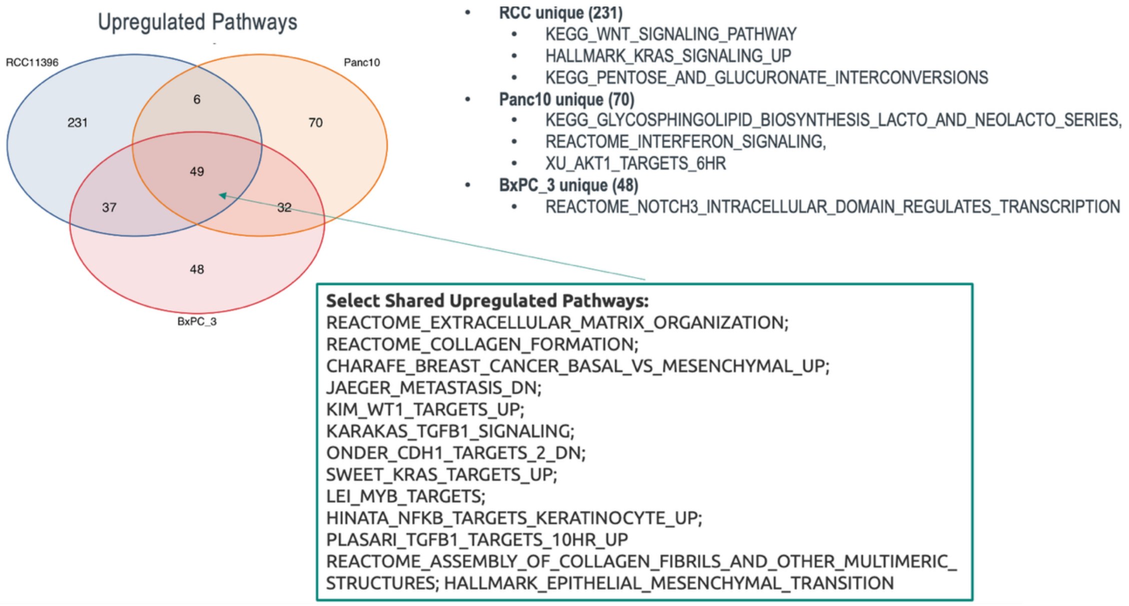

Figure S19. Pathway enrichments of upregulated genes from CAF-tumor co-cultures using RCC, PANC10, and BxPC3 cell lines. Many key processes enriched in CAFs after co-culturing were shared across cancer cell lines, including ECM organization, collagen assembly, TGFβ signaling, and epithelial to mesenchymal transition.

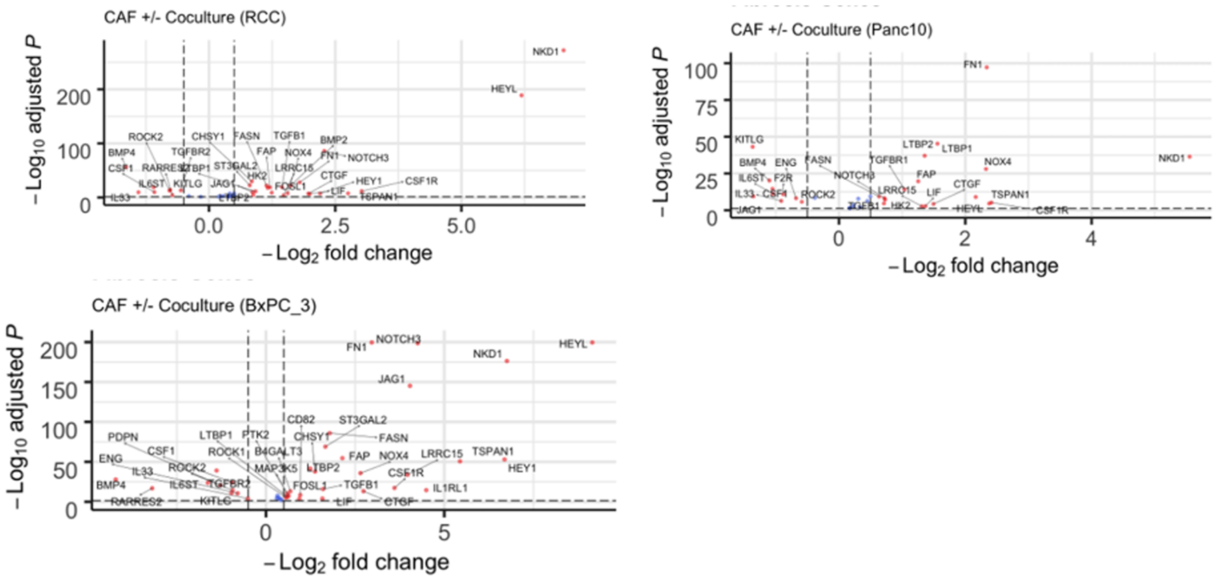

Figure S20. Differentially expressed pan-disease fibrosis genes after CAF-tumor co-culturing. BxPC3-CAF co-culturing resulted in the strongest fold-change in gene expression levels compared to other cell lines.

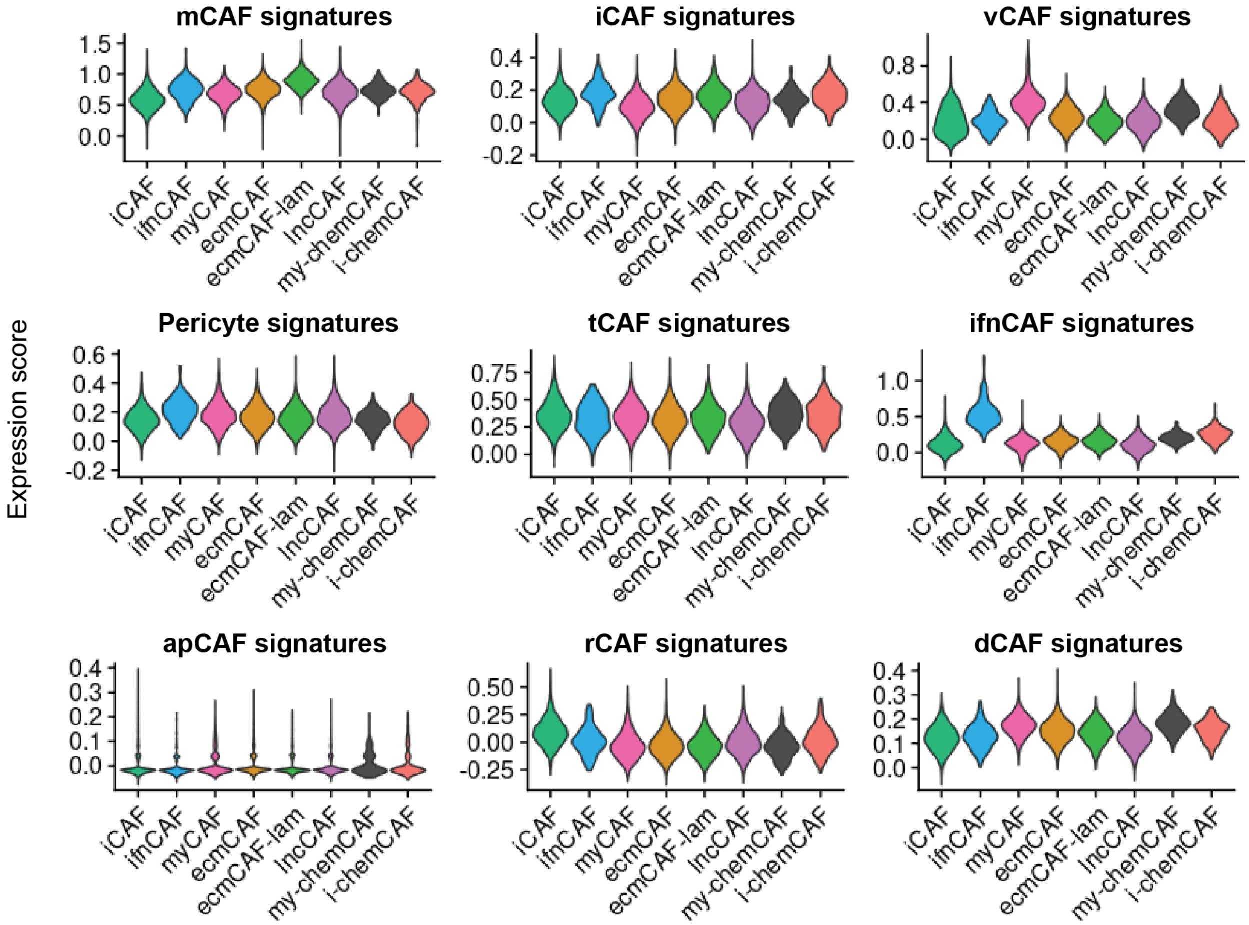

Figure S21. Correlation between *in vitro* CAF subtypes in BxPC3-CAF co-cultures with primary CAF signatures from Cords et al. on primary breast cancer.
